# Intracortical mechanisms of single pulse electrical stimulation (SPES) evoked excitations and inhibitions in humans

**DOI:** 10.1038/s41598-024-62433-0

**Published:** 2024-06-14

**Authors:** Boglárka Hajnal, Johanna Petra Szabó, Emília Tóth, Corey J. Keller, Lucia Wittner, Ashesh D. Mehta, Loránd Erőss, István Ulbert, Dániel Fabó, László Entz

**Affiliations:** 1 Epilepsy Center, Clinic for Neurosurgery and Neurointervention, Semmelweis University, Budapest, 1145 Hungary; 2 János Szentágothai Neurosciences Program, Semmelweis University School of PhD Studies, Budapest, 1083 Hungary; 3 Department of Functional Neurosurgery, Clinic for Neurosurgery and Neurointervention, Semmelweis University, Budapest, 1145 Hungary; 4 Epilepsy and Cognitive Neurophysiology Laboratory, University of Alabama at Birmingham, Birmingham, AL 35294 USA; 5 Department of Neuroscience, Albert Einstein College of Medicine, Bronx, NY 10461 USA; 6 Department of Neurosurgery, Hofstra North Shore LIJ School of Medicine and Feinstein Institute of Medical Research, 300 Community Drive, Manhasset, NY 11030 USA; 7 Department of Neuroscience, Psychiatry and Behavioral Sciences, Stanford University, Palo Alto, CA 94304 USA; 8 Institute of Cognitive Neuroscience and Psychology, Research Centre for Natural Sciences, HUN-REN, Budapest, 1117 Hungary; 9 Department of Information Technology and Bionics, Péter Pázmány Catholic University, Budapest, 1083 Hungary; 10 Lendület Laboratory of Systems Neuroscience, HUN-REN Institute of Experimental Medicine, Budapest, 1083 Hungary

**Keywords:** Neuroscience, Physiology, Neurology

## Abstract

Cortico-cortical evoked potentials (CCEPs) elicited by single-pulse electric stimulation (SPES) are widely used to assess effective connectivity between cortical areas and are also implemented in the presurgical evaluation of epileptic patients. Nevertheless, the cortical generators underlying the various components of CCEPs in humans have not yet been elucidated. Our aim was to describe the laminar pattern arising under SPES evoked CCEP components (P1, N1, P2, N2, P3) and to evaluate the similarities between N2 and the downstate of sleep slow waves. We used intra-cortical laminar microelectrodes (LMEs) to record CCEPs evoked by 10 mA bipolar 0.5 Hz electric pulses in seven patients with medically intractable epilepsy implanted with subdural grids. Based on the laminar profile of CCEPs, the latency of components is not layer-dependent, however their rate of appearance varies across cortical depth and stimulation distance, while the seizure onset zone does not seem to affect the emergence of components. Early neural excitation primarily engages middle and deep layers, propagating to the superficial layers, followed by mainly superficial inhibition, concluding in a sleep slow wave-like inhibition and excitation sequence.

## Introduction

Single-pulse electrical stimulation (SPES) is a widely used diagnostic tool in presurgical evaluation of epileptic patients implanted with intracranial electrodes. Cortico-cortical evoked potentials (CCEPs), especially the ones evoked by low frequency (≤ 1 Hz) SPES, provide information regarding brain network organization^1^ and can aid the localization of pathological areas^2,3^. The term CCEP refers to a reproducible series of putatively physiologic early evoked components, while later deflections (> 100 ms) with variable latencies and inconsistent emergence (repetitive response, delayed response, evoked afterdischarges^4^, etc.) are considered pathologic^5^. However, this strict temporal limit is controversial and there is evidence that late components appear in physiologic conditions, whereas pathologic processes can affect early components^6,7^. Accordingly, epileptic network reorganization can also be captured by the altered pattern of early CCEP responses^8,9^. CCEPs typically consist of an early (N1: 10–50 ms) and a late (N2: 50–500 ms) surface negative deflection^10,11^. While N1, peaking around 10–30 ms, reflects direct cortico-cortical projections within functional networks, N2, with peak between 50 and 300 ms, is thought to be composed by both cortico-cortical and cortico-subcortico-cortical circuit activity, reflecting intra- and internetwork connections^12,13^. Few studies also address P1, the small positive deflection preceding N1, and P2, the one following N1^14^.

Despite the widespread use of cortical electrical and magnetic stimulation, the intracortical mechanisms that generate CCEPs in humans are unknown. Using laminar microelectrodes (LMEs)^15^, there is an opportunity to examine the laminar distribution of sleep slow waves (SW)^16^, epileptiform discharges in human neocortex^17^, and in hippocampus^18^ and also SPES evoked cortical potentials, as we show it in our current study.

SW is defined as the alternation of cortical up and down states during NREM sleep stage 3^19^ and it is widely studied in cats^20^ and also in humans^21,22^. While the active phase (up-state), corresponding to membrane depolarization, is made of excitatory and inhibitory postsynaptic potentials, the inactive phase (downstate) represents hyperpolarization, due to global disfacilitation in the cortico-thalamic network^20^. The contribution of cortico-cortical and cortico-thalamic networks to the generation of SWs has been well-described in sleep in animal models^20^, but it is less understood in humans. Although cortical networks are sufficient to generate SWs—demonstrated in thalamectomized cats^20^, and in cortical slice preparations^23^—it is well known that the thalamus plays an important role in synchronizing populations of cortical neurons^24^. Animal studies report that SWs can be generated in any of the cortical layers, with the highest probability in infragranular cortical layers and with a pivotal role in up-state initiation of layer V pyramidal cells^23,25^. Nevertheless, Zaforas et al. have pointed out in anaesthetized rats that the probability of SWs starting in layer II/III increases in response to spinal cord injury^26^. Interestingly, Csercsa et al. observed the generation of SWs detected in epileptic patients primarily in supragranular layers^16^. Whether the discrepancy between animal and human data arises from the pathologic reorganisation of the epileptic neocortex or from interspecies differences in cortical cell population, is still unclear^27^. SWs appear over large cortical areas due to local intracortical connections^28^ and travel from one definite region to all over the scalp mostly within the default mode network^22,29^. Nevertheless, SWs can also remain local, associated with local cessation of neuronal firing^30^. SWs can be elicited during sleep by cortical stimulation using transcranial electric^31^,-magnetic stimulation (TMS)^32,33^ in humans, and also by intracortical electrical stimulation in rats^34^. In pathological conditions, the synchronized SW downstate in cortex may increase epileptic discharges^35,36^.

In this study, we attempted to determine the intracortical profile of different CCEP components to shed light on the underlying working mode of cortical circuits. Furthermore, as the laminar appearance pattern of late negative component of CCEP is similar to sSW downstate’s (SWd), we hypothesised that evoked negativity represents a cortical downstate that can be electrically triggered also in the awake state.

## Materials and methods

### Patient selection and electrodes

Seven focal epileptic patients (4 males, ages 25.42 ± 11.37 years) undergoing phase 2 presurgical evaluation in our institute (Clinic for Neurosurgery and Neurointervention, Semmelweis University, Budapest, Hungary) were included. Patient characteristics can be found in Table 1. Informed consent was obtained from all subjects and/or their legal guardian(s) according to the declaration of Helsinki and approved by the central ethical committee of our country (National Scientific and Ethical Committee of the Medical Research Council, Budapest, Hungary (Egészségügyi Tudományos Tanács, Tudományos és Kutatásetikai Bizottsága): 242/KO/2001 6008/6/2001/ETT and 20680-4/2012/EKU (368/PI/2012)). All research methods were performed in accordance with the relevant guidelines and regulations**.** The paradigm of cortical stimulation was related to current study and was included in the ethical approval.

**Table 1:** Summary of patients involved in the study.

	Gender	MRI finding	Age at onset	Age at surgery	LME location	Seizure type	Number of missing LME channels	Distance between stimulated and recording electrodes
Pt1	F	Normal	11 y	34 y	R frontal (Th2)	Focal, tonic postural	24	2.5 cm
Pt2	M	R fronto-centro-opercular dysgenesis	5 y	20 y	R frontal	Focal, hypermotor	14, 15, 24	1 cm
Pt3	M	R frontal dysgenesis	5 y	16 y	R frontal (Th2)	Focal, tonic postural	22, 23, 24	1.5 cm
Pt4	F	R frontal dysgenesis	3.5 y	19 y	R frontal	Focal, tonic postural	24	< 1 cm
Pt5	M	R parietal dysgenesis	7.5 y	13 y	R frontal	Focal sensory-motor hemiconvulsive	None	2.5 cm
Pt6	F	R frontal-cingular dysgenesis	24 y	44 y	R frontal (Th2)	Multifocal	22, 23, 24	1.5 cm
Pt7	M	R inferior frontal gy. dysplasia	12 y	32 y	R frontal (Th1)	Focal, hypermotor	24	1 cm

The numbers in parentheses indicate the number of the multielectrode selected for analysis in cases where two electrodes have been implanted.

*F* female, *M* male, *R* right, *y* year, *gy* gyrus.

### Electrode implantation and reconstruction

**Figure 1:**
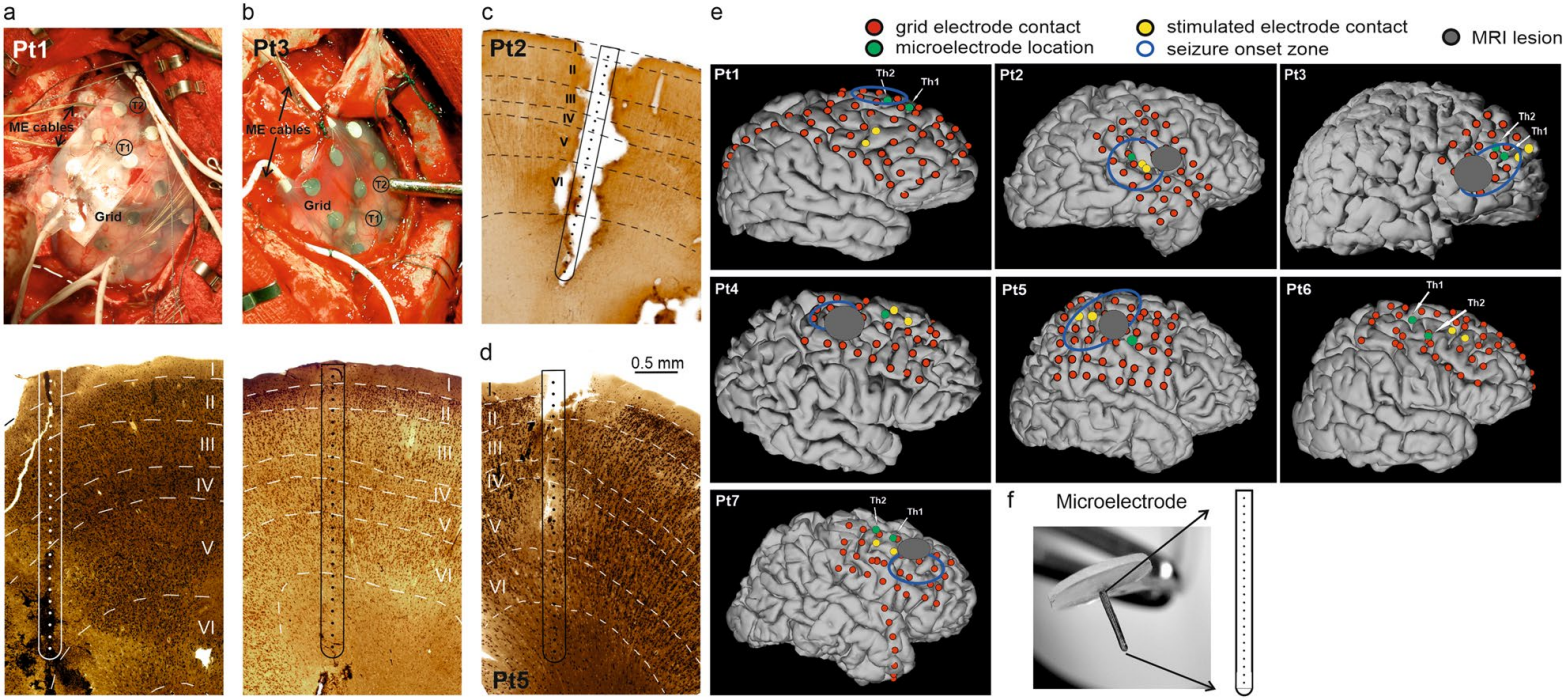
Macro- and microelectrodes. (**a**,**b** row1) Intraoperative photograph of a surface subdural grid electrode and two “thumbtack” multielectrode for LFP and SUA recordings (T1 and T2) in Pt1, Pt3. (**a**,**b** row2, **c**,**d**) Histological reconstruction of electrode penetration track with the position of each microelectrode contact co-registered to the individual cortical layers in Pt1, Pt2, Pt3 and Pt5. Note that all the histologically evaluated electrode tracks verified normal cortical lamination around the electrode trajectory. (**e**) Grid, strip and microelectrode array locations in seven patients included in the study. Surface electrode locations (red filled circle) are superimposed on the 3D reconstructions of preoperative magnetic resonance imaging (MRI) based on postoperative CT scans, locations of microelectrodes (green filled circle) and stimulated electrode contacts with best-response (yellow filled circle) relative to seizure onset zone (blue open circle) and the surface projection of morphologically pathological area on the MRI, i.e. MRI lesion (grey filled circle). Patients 2, 3, 4, 5 and 7 have a pathology of focal cortical dysplasia placed locally deep in sulci and gyri below the marked area. Pt1 is MRI negative and Pt6 has a midline pathology (see Table 1). Note that all the laminar electrodes fell outside the MRI lesion, but inside or very close to seizure onset zone. (**f**) Photo of a 24-contact intracortical laminar multielectrode.

All patients underwent fluoroscopy aided subdural strip and grid implantation (AD TECH Medical Instrument Corp., Racine, WI, USA: various subdural electrodes) planned entirely on clinical grounds. Up to two LMEs (24 contact, 150 μm intercontact distance, 3.5 mm span; Neuronelektród Ltd., Budaörs, Hungary) were inserted perpendicular to the cortical surface underneath the subdural electrodes for scientific purposes^15,37,38^ (Fig. 1a–d). Following electrode implantation, the surface electrode and LME locations were superimposed on the 3D reconstructions of preoperative MRIs based on postoperative computed tomography (CT) scans (Fig. 1e). A gyrus likely to be removed later was selected for microelectrode implantation. After securing the output leads of the LME with appropriate stitches, the subdural grid electrode was positioned above the multielectrode. The electrode penetration track was reconstructed upon removal in the surgical block^16,18,39^ (Fig. 1a–d). Surgery was performed in six out of seven cases. The electrode containing tissue block was removed in 4 patients (Pt.1, 2, 3, 5).

The resected tissue was cut into 2–5 mm blocks (if it was larger, than 5 mm) and immersed into a fixative containing 4% paraformaldehyde, 0.1% glutaraldehyde and 0.2% picric acid in 0.1 M phosphate buffer (PB, pH 7.4). Fresh fixative solution was applied every hour for at least 6 h, while the tissue blocks were constantly agitated. The blocks were post-fixed in the same fixative solution overnight with constant agitation. The next day, 60 µm thick sections were cut from the blocks with a Leica VT1200S vibratome (Leica GmBH, Wetzlar, Germany, RRID:SCR_016495). Photographs were taken from the electrode tracks during the sectioning procedure. Following washing in 0.1 M PB, sections were immersed in 30% sucrose for 1–2 days and then frozen three times over liquid nitrogen. Endogenous peroxidase activity was blocked by 1% H_2_O_2_ in PB for 10 min. Non-specific immunostaining was blocked by 2% normal goat serum and 2% normal horse serum for one hour. Sections containing the electrode track were immunostained against the neuron marker NeuN (1:2000, EMD Millipore, Billerica, MA, USA, RRID:AB_2298772), the astroglial marker glial fibrillary acidic protein antibody (GFAP, 1:2000, EMD Millipore, Billerica, MA, USA, RRID:AB_94844) the pyramidal cell marker non-phosphorylated neurofilament protein SMI-32 (1:4000, Biolegend, San Diego, CA, USA, RRID:AB_2564642) or the perisomatic inhibitory cell marker parvalbumin (PV, 1:7000, Swant, Bellinzona, Switzerland, RRID:AB_10000343). All antibodies were mouse monoclonal antibodies, and applied at 4 °C for 2 days. Their specificity was tested by the manufacturer. Visualization of the immunostained element was performed with the immunoperoxidase method, using biotinylated goat-anti-mouse IgG (1:250 for 2 h, Vector, Burlingame, CA, USA), avidin-biotinylated horseradish peroxidase complex (ABC, 1:250, 1.5 h, Vector, Burlingame, CA, USA) and 3′3-diaminobenzidine, as the chromogene. Sections were osmicated (0.5% OsO_4_ in 0.1 M PB, 20 min), dehydrated in ethanol and mounted in Durcupan (ACM, obtained from Merck, Kenilworth, NJ, USA). Sections were examined and photographs were taken with a Leica bright field microscope (Leica GmBH, Wetzlar, Germany).

The electrode track was reconstructed and the layers of the neocortex were outlined considering all available stained sections. A shrinkage correction factor was applied based on earlier publications^40,41^.

### Surface and laminar recordings

Following implantation, patients were admitted to the epilepsy monitoring unit and continuously monitored for epileptic activity. Video-EEG monitoring was carried out using a standard hospital system (Brain Quick System 2 or 98 or Plus Evolution, Micromed, Mogliano Veneto, Italy). All signals were recorded with mastoid reference (Acquisition rate: 1 or 2 kHz/channel, 16 bit, no filtering) and stored on external hard disks for offline analysis. The data obtained from LMEs were recorded by a separate system consisting of a custom-built preamplifier, amplifier, and a Labview-based acquisition software (National Instruments, Austin, TX), allowing recording of the gradient local field potential (LFP) as a voltage difference between two adjacent electrode contacts. LFP (0.1–500 Hz, 2 kHz/channel, 16 bit) and multiple unit activity (MUA) (500–5000 Hz, 20 kHz/channel, 12 bit) were recorded from the LMEs simultaneously while the patients were awake or sleeping^15^. LME recordings were co-registered to the surface ECoG recordings using a common marker generated by an external trigger computer.

### Single pulse electrical stimulation (SPES) of the neocortex

Cortical stimulation was performed at the bedside on post-implant day 3 to 7. In all cases, before the electrical stimulation sessions, we have already had recorded enough spontaneous seizures for clinical analysis. Stimulation was performed in seizure free periods, at least 4 h after the last recorded seizure. All patients underwent stimulation in the awake state (alert state with eyes opened), verified by video observations and EEG patterns.

SPES (bipolar, 10 mA, 0.5 Hz, biphasic, square pulse, pulse width: 0.2 ms, trial: 20–150) was administered to each adjacent pair of grid and strip electrodes using IRES Surgical 600 stimulator (Micromed S.p.A. Via Giotto, 2-31021, Mogliano Veneto—Italy) (Pt 1, 3, 5) or built in Micromed stimulator from 2015 (Pt. 2, 4, 6, 7). The number of stimuli per pair of electrodes varied from 25 to 150, depending on the patient’s clinical state (stimulation was interrupted if the patient had a seizure during the protocol) and clinical question (considering the relevance of eliciting abnormal CCEPs in presurgical planning). All patients had one stimulation session. Total number of stimulated electrode pairs and stimuli applied for each patient are shown in Supplementary Table S3.

The risk of seizure initiation has been shown to be relatively low when applying 0.5 Hz pulses. Stimulation parameters were selected based on literature data^1,10^ and also our previous experience that 0.5 Hz stimulation above 10 mA does not provide significantly better evoked responses, however it increases the risk of inducing seizures.

### Analysis of sleep SW s and SPES evoked potentials

Data analysis was performed using Neuroscan (Compumedics, El Paso, TX), EEGLAB^42^, and custom MATLAB scripts (MathWorks, Natick, MA). The sleep staging was performed by an expert neurophysiologist (D.F.) based on scalp-EEG and ECoG. The downstate phases of SWs were then detected visually on the corresponding laminar recordings of NREM sleep stage 3. Layer II signal was filtered (low pass 5 Hz, 24 dB/oct, zero-phase shift). Next, exact downstate positions were refined as local minima of the filtered signal. Two second-length epochs (± 1000 ms) time-locked to downstate peaks and averaged for each LME channel were used for spectral, current source density (CSD) and MUA analyses (see sections below).

**Figure 2:**
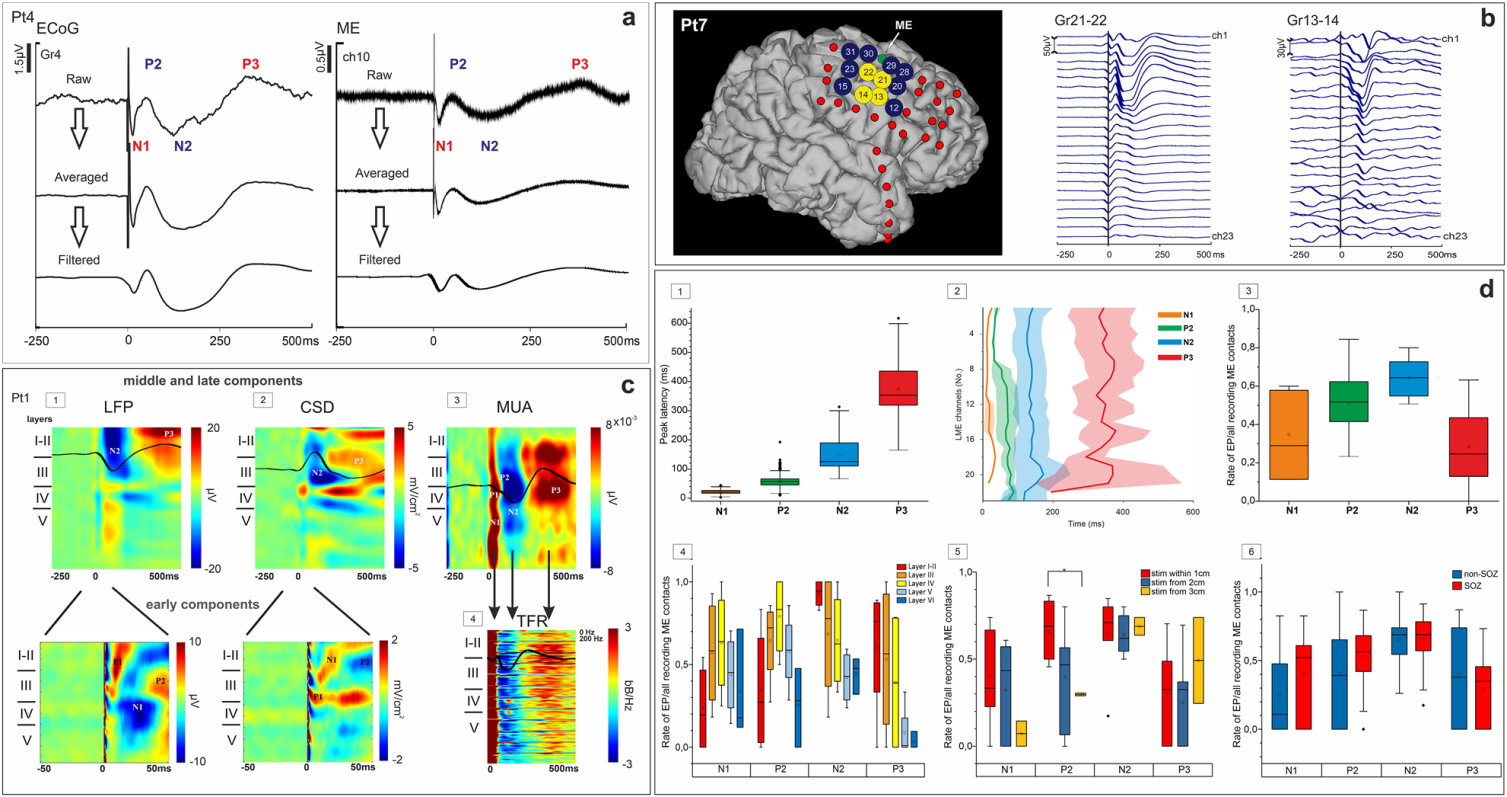
CCEP components. (**a**) Components of CCEPs are consistent, regardless of signal processing method (i.e. averaging/ low pass filtering), both on ECoG and LME recordings. (**b**) Single-trial example (Pt7) of early components triggered from the vicinity of LME (Gr21-22) and of middle and late components triggered from more distant contacts (Gr13-14). (**c**) Laminar profile of CCEPs illustrated by the recordings of Pt1 (averaged across trials of a single stimulation site), aligned to stimulation: LFP data (**c/1**), CSD (**c/2**), MUA (**c/3**), TFR over 0–200 Hz (calculated for each channel, plotted on top of each other; **c/4**). (**c/1**) and (**c/2**) bottom row: zoomed in to make early components more visible. Note that zoomed-in figures are unfiltered (only notch filter is applied to remove power noise) to make P1 component visible, while those in the top row are filtered with 20 Hz (48 dB). White and black curves mark the activity on the max-amplitude channel. The early components consist of a surface positivity with lower-middle layer sink (P1) and surface negativity with superficial sink (N1), accompanied by elevated MUA. The middle components consist of surface positivity (P2) with superficial source and surface negativity (N2) with wide middle layer source, accompanied by marked decrease in MUA. P3 is characterised by surface positivity with middle layer sink and upper-middle layer increase in MUA. The spectral power shows increase during early and late components and decrease during P2 and N2. (**d**) Peak latencies of LFP components (n = 7): pooled over all LME channels (mean, median, 1st and 3rd quartiles; **d/1**); across LME channels with no significant depth-dependent change (**d/2**). Considering the total number of detected components, N2 is evoked at the highest rate (n = 7, **d/3**). Although statistically non-significant, N1 and P2 components tend to be evoked in middle-, while N2 and P3 in supragranular layers (n = 4, **d/4**). The appearance of components is distance-dependent: N1 and P2 are most likely evoked within 1 cm from LME, P3 rather from a distance and N2 both up close and from further away (n = 7, **d/5**). There are no significant differences between stimulation from SOZ vs. non-SOZ, regarding appearance of components, though there is a tendency that N1 and P2 can be better evoked from SOZ (n = 7, **d/6**) (see “Results” for details of statistics).

Evoked responses to stimulation were divided in 1250 ms epochs (250 ms pre-stimulation to 1000 ms post-stimulation) time-locked to stimulation pulse delivery. Following low-pass filtering (20 Hz, 48 dB/oct) and baseline correction (− 250 to − 50 ms), the raw LFP signals were averaged in each grid pair stimulation trial. CCEP components’ peak amplitude was measured as the absolute peak voltage between 5 and 500 ms. Z-score was computed using the standard deviation (SD) of baseline (− 250 to − 50 ms). Evoked potential latency was defined as the time from stimulus to peak of each component. The ratio of each detected component and the total recording on LME channels for each patient were determined, including all grid stimulation sites that triggered LME responses. The ratio of triggerability of individual components by layer and by distance from stimulated grid contacts, was also analysed (Fig. 2d).

**Figure 3:**
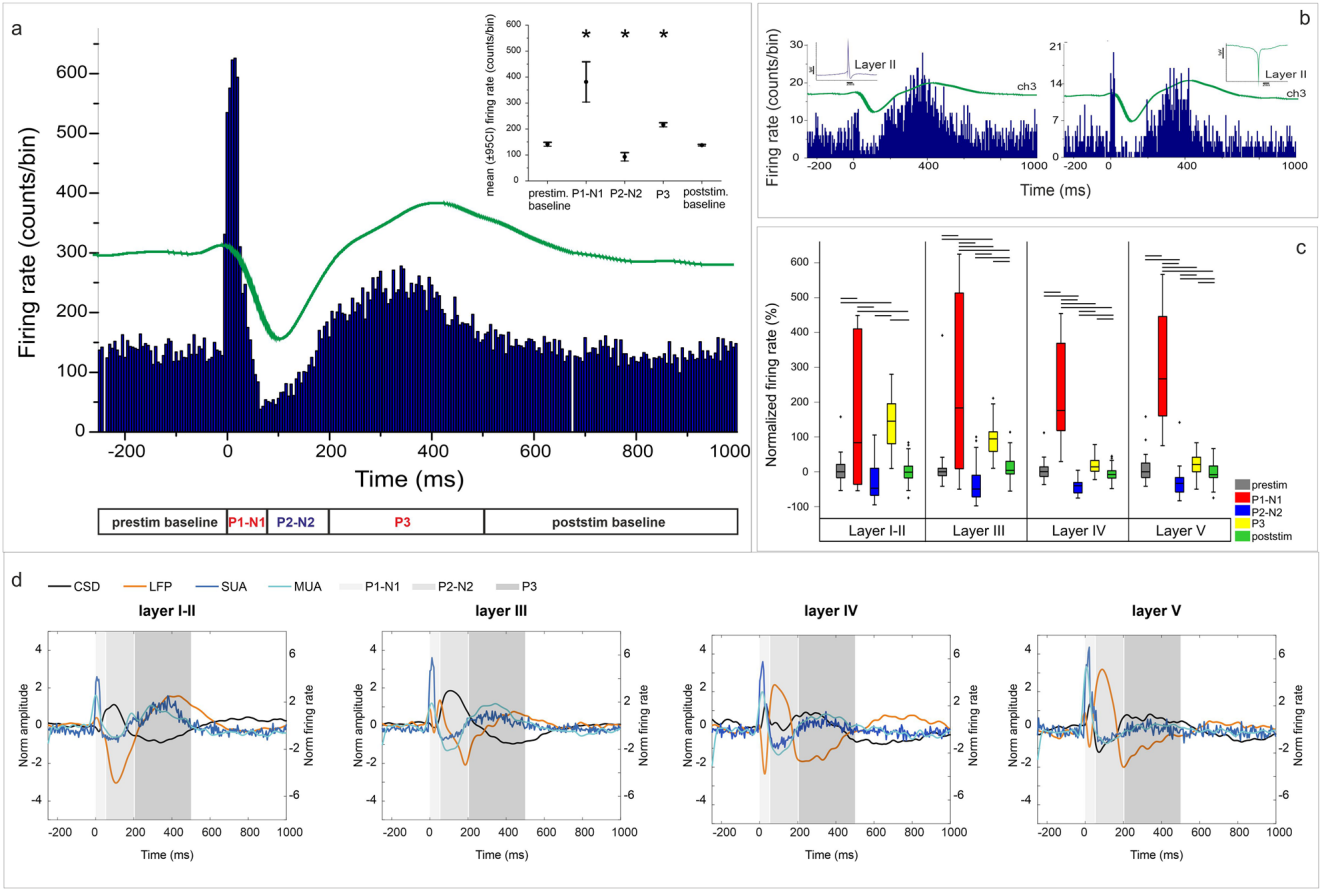
Single unit correlates of CCEPs. (**a**) Time histogram of CCEP-related firing activity, averaged across stimulation trials. The firing rate of clustered cortical neurons (n = 26, Pt1) shows significant and sequential changes after electrical stimulation. While in the time-window of P1-N1 components (0–50 ms) and during P3 (205–500 ms) there is a statistically significant increase in cell firing rate, during P2-N2 components (55–200 ms) the neuronal firing decreases significantly compared to the SUA measured both in pre- and poststimulation intervals (− 250–(− 5 ms) and 500–1000 ms, respectively). Asterisks on error bar plots indicate statistically significant differences in firing rate between all CCEP intervals and also between each interval and pre-and poststim. baseline periods (Kruskal–Wallis ANOVA; χ^2^(4) = 151.8, p < 0.001. Mann–Whitney U post-hoc tests, p < 0.01). (**b**) Changes in firing rates of two individual neurons recorded from layer II. (**c**) The most prominent changes in cell firing rate relative to prestimulus window are seen in layer V during P1-N1, although there is a statistically significant change in SUA in all cortical layers (significant differences, with p < 0.001, are marked by lines above boxplots, one-way ANOVA with Bonferroni post-hoc tests, for details see “Results”). Boxplots represent median with interquartile range (IQR), whiskers mark the most extreme values within 1.5xIQR. Crosses mark outliers outside this range. (**d**) Overlay plots of LFP (orange), CSD (black), MUA (light blue) and SUA (dark blue) in Pt1, separated layer-by-layer (averaged across channels within the corresponding layer). Shaded areas represent the same time-windows as in case of (**a**). Note that negative CSD values represent sinks, while positive values mark sources.

**Figure 4:**
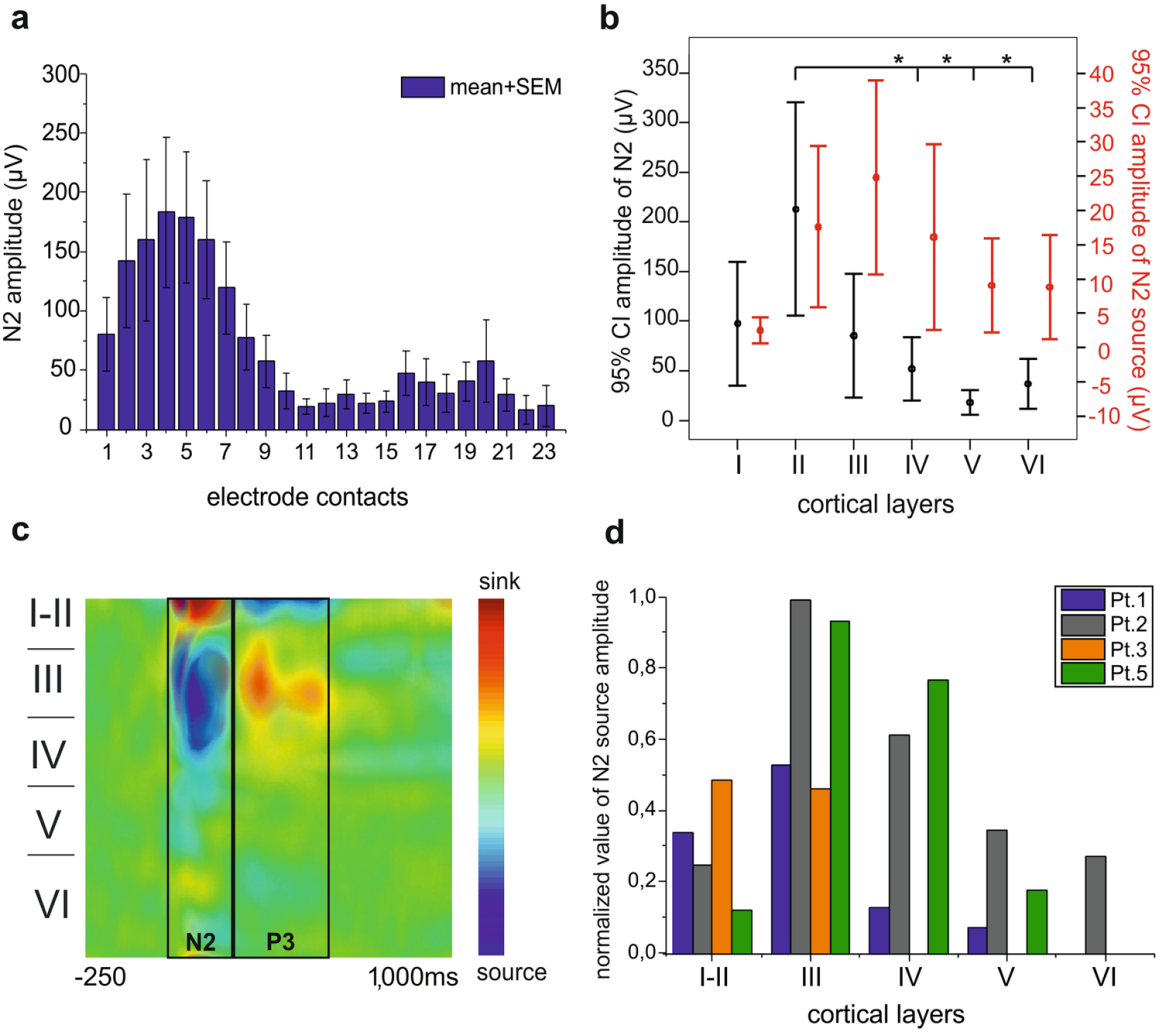
Laminar distribution of N2 LFP, current sinks and sources. (**a**) Changes of N2 field potential gradient amplitude across microelectrode contacts and averaged across trials (within each subject), in 7 patients. (**b**) In 4 patients with histological reconstruction, there are significant differences in N2 amplitude between cortical layers as revealed by one-way ANOVA (F(5) = 7.7, p < 0.001, η^2^ = 0.336) and Tukey HSD post-hoc test (t_II-IV_ = 4.3, p < 0.001, t_II-V_ = 5.77, p < 0.001, t_II-VI_ = 4.89, p < 0.001) (black bars). There was no significant difference in N2 – CSD source amplitude (indicated by red bars) between cortical layers. (**c**) The biphasic distribution of current sinks and sources of CCEPs across cortical layers. Overlay of 6 patients. (**d**) The N2 current source is maximal in middle cortical layers around layer III. Data is normalised to the peak response in each patient.

**Figure 5:**
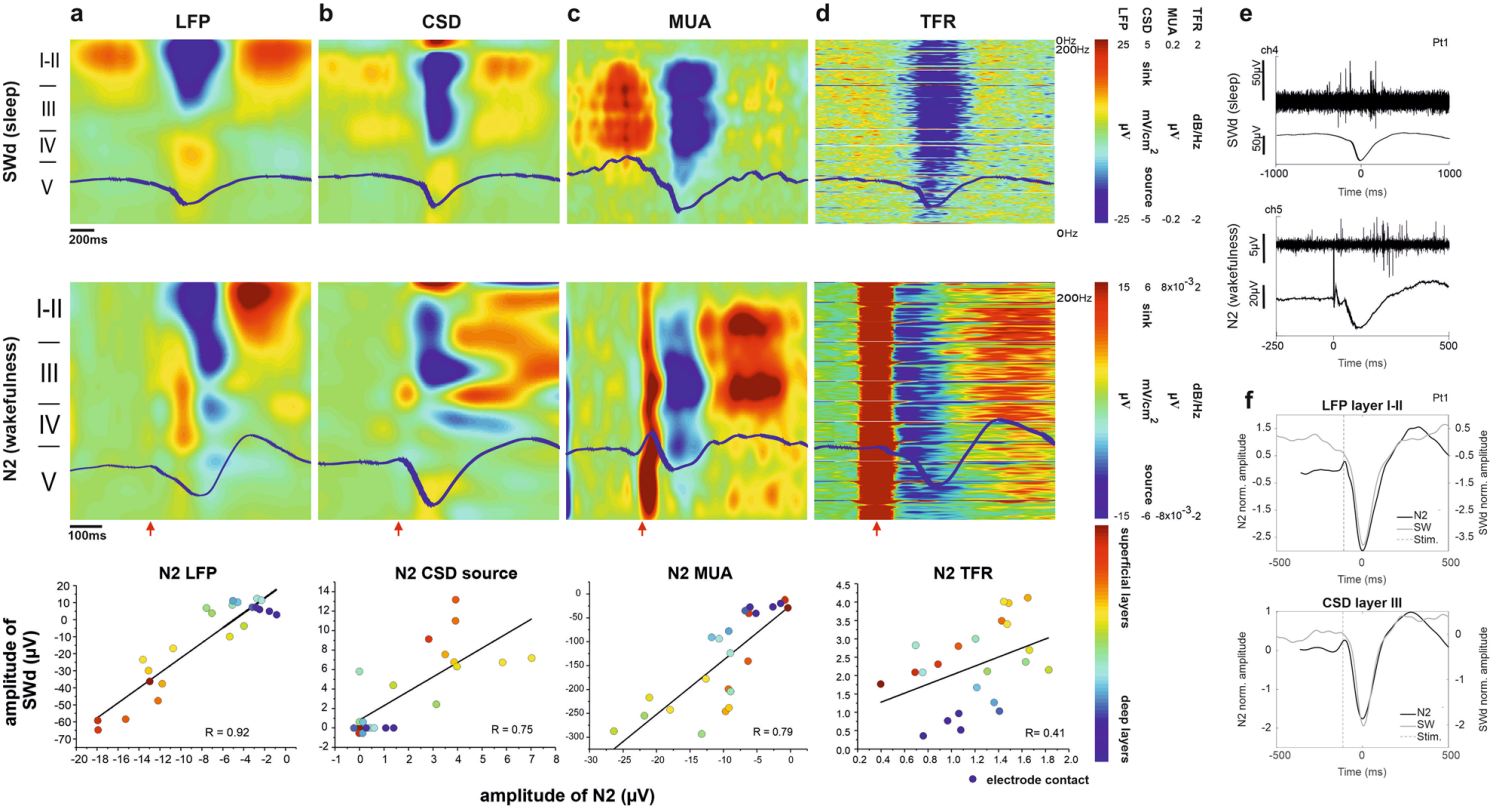
Electrically induced N2 during awake state is similar to the downstate of spontaneous slow waves (SWd) during sleep. (**a**) LFP; (**b**) CSD; (**c**) MUA; (**d**) TFR. **Row 1**: Laminar profile of the spontaneous SW time locked to the downstate (trial-average of Pt1). The downstate is characterised by upper layer surface negativity (**a**), a middle layer source (**b**) accompanied by a marked decrease in multiunit activity (**c**) in the same layers, and a broadband power decrease (**d**) through all cortical layers. **Row 2**: Laminar analysis of CCEP in awake state (trial-average of Pt1). LFP shows a superficial negativity during the N2 (**a**). CSD distribution shows an upper-middle layer source during the time of N2 (**b**). MUA is decreased during N2 in middle cortical layers (**c**). The TFR shows a wide-band spectral power decrease through all cortical layers during N2 (**d**). Note the time scale differences between the two analyses (− 250–1000 ms for the N2 and − 1000–1000 ms for the SWd). Red arrows indicate the time of stimulus. **Row 3**: Statistical comparison of SWd and N2 characteristics based on Pearson’s r correlation method (For LFP r = 0.92, CSD source r = 0.75, MUA r = 0.79, TFR r = 0.41, p < 0.05). The different color dots indicate individual electrode contacts. The warm color dots are for contacts located in superficial layers, the cold color dots indicate electrodes from deep layers (see color bar). The zero value means there is no detectable SWd or N2 component on that channel. All data are derived from Pt1. (**e**) Averaged SWd (top) and N2 (bottom) waveforms (Pt1) on selected channels (marked in left corner of the plots) of low-pass filtered LFP and associated MUA recordings presenting the characteristic period of neural silence. Note the different amplitude and time scales applied in case of averaged waveform plots. (**f**) Overlay of SWd (grey curves) and N2 (black curves) averaged waveforms (Pt1). LFP (top) is derived from layer I-II, while the CSD (bottom) from layer III recordings (corresponding to the layers of maximal event amplitudes). Stimulation preceding N2 is marked with a dashed line.

**Figure 6:**
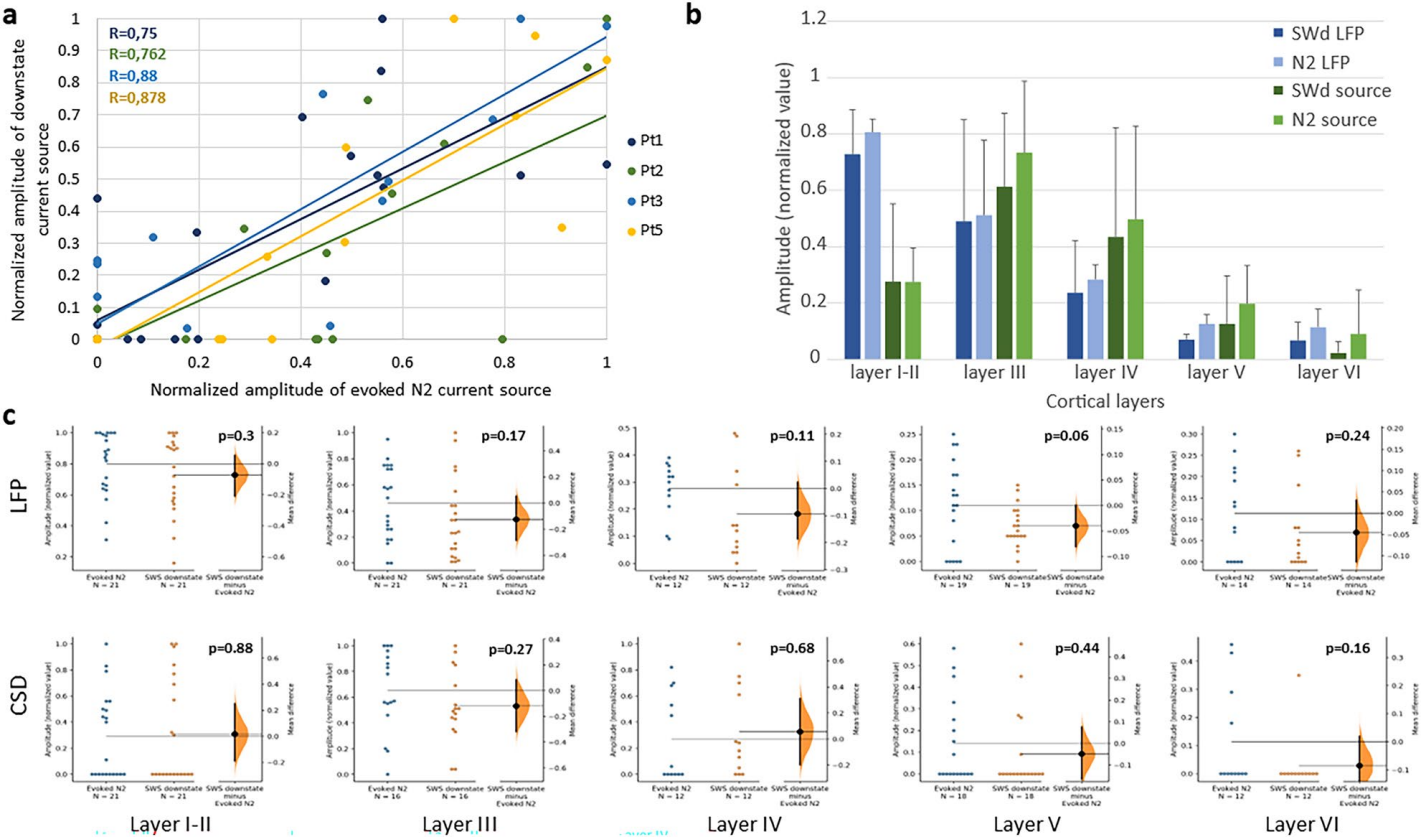
Electrically evoked N2 and downstate of spontaneous slow waves (SWd) show a similar appearance pattern in cortical layers. (**a**) Scatter plots show a strong correlation between current source amplitudes of SWd and N2 in all four patients (for Pt1 Pearson’s R = 0.75, Pt2 R = 0.76, Pt3 R = 0.88, Pt5 R = 0.878). The color dots indicate individual electrode contacts. Amplitude values of 0 mark cases where N2/SWd were not detectable. The dots in 0–0 locations represent more than one electrode contact per patient (for Pt 1 = 6, Pt2 = 9, Pt3 = 8, Pt5 = 11 contacts). (**b**) Averaged normalised field potential and current source amplitudes of SWd and N2 in each cortical layer. There is no significant difference in amplitude between N2 and SWd in either cortical layer, both in terms of field potentials and current sources (p > 0.05; two-sample t-tests for each cortical layer). Maximal amplitudes are measured in supragranular cortical layers (field potential: layer I-II, current source: layer III). Data are derived from the four patients (Pt1,2,3,5) with histological reconstruction of electrode penetration track. Error-bars represent standard deviation. (**c**) Estimation statistics on distribution of N2 and SWd by cortical layers show non-significant differences. The mean difference between N2 and SWd is shown in Gardner-Altman estimation plot. Both groups are plotted on the left axes; the colored dots represent the individual electrode contacts; the mean difference is plotted on floating axes on the right as a bootstrap sampling distribution. The mean difference in normalised amplitude values and 95% confidence interval is indicated. Note that none of the p values indicate statistical difference.

LFP, CSD, MUA, TFR, SUA analyses (Figs. 2c, 3, 4, 5, 6) and peak latency across channels (Fig. 2d/2) were examined in stimulation sites yielding the highest amplitude response (see Fig. 1e, yellow dots, for the location of preferred stimulation sites) on signals averaged across stimulation trials. In case of measuring peak latency pooled across channels and patients (Fig. 2d/1) and rate of evoked components (Fig. 2d/3–6) all stimulated sites with visible CCEP were included.

When examining the layer-by-layer distribution of CCEP N2 and SW downstate, amplitude values (Figs. 4d and 6a–c and Supplementary Fig. S5) of each patient were normalised across LME channels.

### Wide-band spectral power, current source density (CSD), multiple unit activity (MUA) and single unit activity (SUA) analysis

Following removal of stimulated channels and bad epochs (> 500 µV: − 250 to − 10 ms or 50–1000 ms), baseline correction (− 250 to − 50 ms) was applied. Time–frequency response (TFR) analysis for spontaneous and stimulation-induced oscillatory power changes between 1 and 200 Hz was computed and visualised on event-related spectral perturbation (ERSP in dB) maps^42^ for each LME channel, in case of 2 patients (Pt1, Pt2), where the quality of the recordings were sufficient to obtain meaningful measures. Single-trial ERSPs were averaged and plotted on depth-versus-time colour maps. Significant decrease and increase in ERSP was marked with colour blue and red, respectively (see below for details regarding statistical analysis).

CSD analysis^43^ was done by second spatial derivation of LFP trial-averages after spatial hamming-window smoothing (detailed by Ulbert et al.^15^) and filtering (low pass, 20 Hz, zero phase shift, 24 dB/oct). In our case the raw LFP signal was inherently the first spatial derivative of the field potentials, since it was recorded as gradual bipolar montage between contacts along the laminar electrode’s shaft (Supplementary Fig. S4). We applied a second derivation to get the CSD signal: positive values are present where summed transmembrane currents exit the neighbouring neurons (source), while negative values are measured where current enters the neurons (sink). One-dimensional averaged CSD data are presented on depth-versus-time maps, with colour—coded sink (red) and source (blue) amplitudes. CSD was calculated for each patient (n = 7).

MUA was calculated using high frequency filtering (1) 500–5000 Hz, zero phase shift, 48 dB/oct; (2) full wave rectification; (3) low pass filter: 20 Hz, 24 dB/oct) on individual sweeps. The sweeps, averaged across epochs, are visualised on depth-versus-time maps as colour-coded activity increase (red) and decrease (blue) in population activity. The recording quality allowed for MUA calculation in case of 2 patients (Pt1, Pt2).

Single-unit activity (SUA) analysis was performed on LME data recorded with high sampling rate (20 kHz) in case of one patient (Pt1), where individual cell activities could be separated reliably from MUA/field activity and background noise. SUA was detected on epoched raw LME data sampled at 20 kHz (from − 250 to 1000 ms relative to stimulation start). After DC offset removal, we detected SUA with an amplitude threshold adjusted manually according to the magnitude of background noise of each channel. Multiple individual neurons were identified as the generators of SUA on each channel based on clustering with on-line template setup by action potential morphology and amplitude in a 0.4 ms timeframe. We applied a principal component analysis based on template waveform correlations to refine clustering and reduce false detections. Then each cluster was revised visually. All SUA detections were performed in Spike2 (version 7 software (CED Limited, UK). All SUA detections were pooled from all cells on the same channel and from all LME channels in the same layer, resulting in time histograms of CCEP-associated single-unit firing. Histograms of single trials were then averaged across stimulation epochs corresponding to one stimulated channel-pair. Representative examples of autocorrelation of clustered units are shown in Supplementary Fig. S6.

### Statistical analysis

Statistical analyses were performed using IBM SPSS (version 16.0), JASP (version 0.18.3), jamovi (version 1.6.15.0) softwares and in-built Matlab (2019b) algorithms. Normality of distributions were tested with the Kolmogorov–Smirnov test. If normality was rejected non-parametric tests were applied, except for some cases (rate of appearance of CCEP components, Fig. 2d/3–4), when there were only moderate deviations from normality, acceptable in case of one-way ANOVA. To test the temporal consistency of CCEP components across cortical depth we calculated peak latencies of each component for each LME channel (7 patients) and used one-way ANOVA to test the significance of differences between LME channels (Fig. 2d/2).

To compare the rate of evoked potentials across LME contacts (7 patients, Fig. 2d/3) and cortical layers (4 patients, Fig. 2d/4), we have applied one-way ANOVA for each CCEP component). Rates of CCEPs were also compared according to stimulation distance (within 1, 2 or 3 cm from CCEP presenting LME contact), using Kruskal–Wallis with post-hoc Tukey–Kramer tests (Fig. 2d/5), and to stimulation site [seizure onset zone (SOZ) vs. non-SOZ], using Mann–Whitney U test (Fig. 2d/6).

The firing rate (FR) of cortical neurons (n = 26) of one patient (Pt1) was compared across various time intervals: pre- (− 250 to − 5 ms) and poststimulation (500 to 1000 ms) baseline periods and time intervals corresponding to different CCEP components. First, units from all LME channels were pooled and the changes of FR in time were compared by Kruskal–Wallis ANOVA and Mann–Whitney U post-hoc tests (Fig. 3a). Then additional statistical tests were performed for each cortical layer separately, using one-way ANOVA with Bonferroni post-hoc tests (Fig. 3c).

We have analysed in more details the intracortical laminar profile of N2 components across patients. For this purpose, we averaged the absolute value of LFP amplitude at each LME channel for N2 (Fig. 4a) and LFP and current source amplitude at each cortical layer, in four patients (Pt1, 2, 3, 5), where histology was available allowing precise localization of microelectrode contacts to cortical layer (Fig. 4b,d). To test the statistical significance of differences in absolute amplitude values (both for LFP N2 and N2 current source) between cortical layers (and also between supragranular layers: layer I-III and granular-infragranular layers: layer IV-VI), we applied one-way ANOVA with Tukey HSD post-hoc test and Kruskal- Wallis ANOVA with Dwass-Steel-Critchlow-Fligner post-hoc analysis, following the correction of significance level according to the number of comparisons (significance level: p < 0.05, corrected significance level: p < 0.0033). Results are shown on whisker plot [dot = mean; whisker = 95% confidence interval; asterisk = statistically significant difference] (Fig. 4b).

We have compared the characteristics of sSW and N2 components, using LFP and CSD data of four (Pt 1,2,3,5) and MUA and TFR data of two patients (Pt 1,2) (Fig. 5, Supplementary Fig. S4). Pearson’s r correlation method was used to evaluate the relationship between SW and N2 measures with p < 0.05 significance level criterion, and we presented the results on scatter plots [dots = channels of LME] (Fig. 5 row 3 and Fig. 6a). The significance of difference in normalised amplitude values between N2 and SW downstate was determined by two-sample t-tests for each cortical layer (Fig. 6b), and confirmed by two sided permutation t-test (Gardner-Altman estimation statistics plots) (Fig. 6c)^44^.

Due to low sample size, Bayesian statistics were also applied: Bayesian ANOVA for the layer-by-layer and distance-dependent comparison of components and Bayesian Mann Whitney U test for the comparison of appearance rates in SOZ vs non-SOZ areas. To define the strength of evidence for correlation between N2 and SW downstate amplitude values in each cortical layer we performed Bayesian Pearson correlation (Supplementary Table S4). The summary of the used statistical tests can be found in the Supplementary Table S4.

## Results

Clinical subdural grid and strip electrodes were implanted over the frontal, parietal and temporal cortices in seven patients to localise both the epileptogenic and eloquent cortical areas (Fig. 1e). Six patients had single focus (frontal n = 5; parietal n = 1; for detailed types of seizure onset see Table 1). One patient (Pt6) had multifocal seizure onset, therefore resective surgery was contraindicated in this case. LMEs (Fig. 1f) were implanted into the frontal lobe, underneath the subdural electrodes, in all seven cases (Fig. 1a,b). No clinical side effects or complications related to the multielectrodes were observed. Patients 2, 3, 4, 5 and 7 had a pathology of focal cortical dysplasia placed locally deep in sulci and gyri below the area marked by grey circles on Fig. 1e. Pt1 was MRI negative and Pt6 had a midline pathology (see Table 1). All the laminar electrodes fell outside the MRI lesion, but inside or very close to seizure onset zone (Fig. 1e).

In four cases (Pt1, 2, 3, 5), histological reconstruction of the neocortical laminae was performed along the penetration track of the removed multielectrode (Fig. 1a–d). Supplementary Table S1 shows LME channels corresponding to cortical layers in these patients. Electrode penetration tracks (Fig. 1a–d) showed intact cortical laminarization (well-preserved pyramidal cells, interneurons and glias, indicating no structural damage of the examined cortex). The thumbtack electrode successfully penetrated all six layers in three cases, while in one case (Pt1) the tip of the microelectrode ended in layer V. In the remaining cases (3/7), cortical areas sampled by LMEs were not removed according to the surgical plan.

### Characteristics and laminar distribution of CCEP components

CCEPs were recorded both with surface and laminar electrodes in seven patients (Fig. 1e). Characteristic components of CCEPs were identified and analysed: early (P1, N1), middle (P2, N2) and late (P3) evoked responses (Fig. 2a, Supplementary Fig. S1), with special emphasis on N2.

The appearance of P1 was less consistent compared to other components (it was found in only 3/7 patients) and was highly dependent on stimulation distance, mostly visible on adjacent surface electrodes (Fig. 2b, Supplementary Fig. S1). Components of CCEPs could be clearly identified and were consistent, regardless of different signal processing methods (i.e. averaging and low pass filtering), both on ECoG and LME recordings, except for P1 which tended to smear with low-pass filtering (Fig. 2a, due to its inconsistent appearance of P1, it is not shown in this figure). The appearance of some components is distance-dependent, i.e. the early ones (P1, N1) can be triggered mostly from the vicinity of LME, while the middle and late ones (P2, N2, P3) can also be triggered from a more distant site (Fig. 2b and d/5).

We have analysed the components using LFP and CSD data of seven, and using MUA and TFR data of two patients, in a time-window of − 250 to + 500 ms relative to stimulation. The resulting activation patterns were qualitatively very similar in all patients (Figs. 2c, 4c, 5 row 2, Supplementary Figs. S1, S2) and revealed different intracortical mechanisms in the background of CCEP components. Figure 2c shows the typical LFP, CSD, MUA and TFR patterns. LFP under P1 exhibits a maximal positivity and a sink-source pair located around cortical layer III, while there is a considerable sink also in deeper layers. It is associated with increased MUA and TFR, encompassing all cortical layers. The N1 component appears as a negative wave in layer III LFP and is linked to a sink in cortical layers I-II, and source pattern reaching to deeper layers, with increased MUA and TFR, mainly in infragranular layers. The prominent positive wave seen during P2 in the LFP engages multiple layers and is associated with a source in cortical layers I–II, and decreased MUA and TFR power. The N2 shows the most marked negative deflection in layers I–III, while it is accompanied by a source in cortical layers III–IV with decreased MUA and TFR power during the descending phase across all layers (see more detailed description of N2 in next section). This LFP pattern is subsequently inverted (positive deflection) in upper layers during P3 component, which corresponds to a sink at layers III-IV concomitant with increased MUA and TFR in layers I–IV. MUA and TFR increase already starts at the ascending phase of N2.

It has to be noted that LFP, CSD, TFR and MUA were only analysed in preferred stimulation sites, i.e. those yielding the highest amplitude response. Nevertheless, the pattern obtained in non-preferred stimulation sites was similar to that presented above, although with lower signal-to-noise ratio (Exemples for CSD patterns corresponding to preferred and non-preferred sites are presented in Supplementary Fig. S2). We have chosen not to include a more detailed analysis of the cortico-cortical connectivity revealed by CCEPs^1,45^, which would imply the more thorough examination of the non-preferred stimulation sites, because we consider it would be out of the scope of this study, primarily focused on the laminar organisation of SPES-evoked components and their relationship with sleep phenomena. Nevertheless, as this would add valuable insight into CCEP-related mechanisms on a network level, this might be addressed in a separate study, including a higher sample size.

The peak latency of evoked components was measured on individual LME contacts. Latencies derived from all stimulation epochs with elicited responses and averaged across LME contacts and patients were, as follows (mean ± SD): N1 21.95 ± 7.16 ms, P2 57.47 ± 20.7 ms, N2 147.31 ± 51.7 ms, P3 374.88 ± 83.22 ms (Fig. 2d/1, n = 7). No significant difference was observed in latencies across cortical depth, as shown by the results of one-way ANOVA: N1 F(20) = 0.32 and p = 0.96; P2 F(22) = 1.3 and p = 0.24; N2 F(22) = 0.36 and p = 1; P3 F(21) = 0.33 and p = 1 (Fig. 2d/2, n = 7). In this case only the epoch with the best response was used for each patient.

The rate of appearance of each component was calculated considering all the CCEP-eliciting stimulations of all patients (mean ± SD): N1 0.35 ± 0.2; N2 0.64 ± 0.1; P2 0.51 ± 0.2; P3 0.28 ± 0.2. The highest rate was observed in case of N2 (Fig. 2d/3, n = 7), which was significantly greater than the rate of N1 and P3 (one-way ANOVA p = 0.0008, F(3) = 7.48; post-hoc Tukey–Kramer). When dividing occurrence rates to cortical layers (n = 4, with histological reconstruction), N1 and P2 components appeared to be more frequent in middle (III-IV), while N2 and P3 in superficial (I-II-III) layers (Fig. 2d/4, n = 4), although these observations were statistically not significant (one-way ANOVA N1: p = 0.388, F(4) = 1.116, P2: p = 0.124, F(4) = 2.183; N2: p = 0.081, F(4) = 2.606; P3: p = 0.182, F(4) = 1.813). In both cases, normal distributions were tested with Kolmogorov–Smirnov test (p-values shown in Supplementary Table S4). Bayesian ANOVA showed only with anecdotal evidence that the rate of appearance of N1 and P3 components is not different across layers, and also with anecdotal evidence that the rate of appearance of P2 and N2 might be different across layers (see detailed statistical results in Supplementary Table S4).

CCEPs elicited from varying stimulation distance contained different components at varying degree. N1 and P2 components were triggered most likely from within 1 cm, P3 emerged most often within 3 cm distance, while N2 seemed to be independent from stimulation distance (Fig. 2d/5, n = 7). These observations were confirmed by statistical testing only in the case of P2 evoked from 1 vs 3 cm (Kruskall–Wallis test, post-hoc Tukey–Kramer N1: p = 0.1457, χ^2^(2) = 3.85; P2: p = 0.01947, χ^2^(2) = 7.88; N2: p = 0.68, χ^2^(2) = 0.77; P3: p = 0.4185, χ^2^(2) = 0.41) The result of Bayesian ANOVA show only anecdotal evidence also in this case: in favour of no distance-dependence in case of N1, N2 and P3 and in favour of distance-dependence in case of P2 ( for detailed statistical results see Supplementary Table S4). Normality of distributions was rejected in most cases, tested by Kolmogorov–Smirnov test (p values in Supplementary Table S4). Figure 2b illustrates this by an example where early components are visible only following stimulation of adjacent electrodes, while later components emerged by the stimulation of more distant contacts.

Whether a certain component can be triggered more often when stimulated from the SOZ, compared to non-SOZ areas, has not been confirmed by our analysis (Fig. 2d/6, n = 7). Although N1 and P2 components seem to emerge at a higher rate in case of SOZ stimulation, the difference was statistically not significant (Mann–Whitney U-test N1: p = 0.3681, U = 268.5; P2: p = 0.80444, U = 149; N2: p = 0.90358, U = 570; P3: p = 0.05152, U = 206.5 while the evidence provided by Bayesian Mann–Whitney U test was only anecdotal (for detailed statistical results see Supplementary Table S4). Normal distribution was rejected by Kolmogorov–Smirnov test (p values in Supplementary Table S4).

### Single unit correlates of CCEP

Analysis of SUA was performed in the case of one patient, with the highest quality recording (Pt1), resulting in 26 clustered cells. The nr. of single unit clusters corresponding to each cortical layer is presented in Supplementary Table S2. The firing pattern of detected neurons was examined during the time-windows of different CCEP components. Significant changes relative to pre- (from − 250 to − 5 ms) and poststimulation (500–1000 ms) periods were found (Kruskal–Wallis ANOVA; χ^2^(4) = 151.8, p = 0.0001, Mann–Whitney U post-hoc tests, p < 0.01). During P1 and N1 (0–50 ms), the firing rate of neurons increased significantly, followed by a significant decrease occurring in the time-window of P2 and N2 (55–200 ms). P3 window (205–500 ms) was again associated with increased firing activity (Fig. 3a,b).

The same pattern was shown to be significant in all cortical layers (one-way ANOVA with Bonferroni post-hoc tests; layerI–II: F(4) = 67.47, p = 2*10^–38^; layerIII: F(4) = 51.42, p = 3*10^–39^; layerIV: F(4) = 129.21, p = 4*10^–54^; layerV: F(4) = 115.17, p4*10^–55^), although the changes relative to prestimulation window were most prominent in case of layer V (Fig. 3c). Temporal association of spiking activity, MUA, CSD and LFP has been visualised by overlaying these time series (Fig. 3d).

### Laminar distribution of N2 component

Intracortical amplitude distribution of N2 is shown in Figs. 4a,b, 5a row2, 6b and Supplementary Figs. S4, S5. LFP under N2 of 7 patients showed negative deflection with amplitude maxima in the upper channels (Fig. 4a, n = 7). N2 amplitude of the 4 patients with histological reconstruction was significantly greater in supragranular (I-III) versus infragranular (IV-VI) layers (0.7 versus 0.15, grand average for baseline normalised values, Mann–Whitney U-test (U = 452, p = 0.0001). The greatest amplitude was observed in layer II (Bonferroni adjusted at α = 0.0033). Layer II showed higher amplitudes than layer IV (t(5) = 4.3, p = 0.001), layer V (t(5) = 5.77, p = 0.001) and layer VI (t(5) = 4.89, p = 0.001) (one-way ANOVA and Tukey HSD post-hoc test with Bonferroni correction (F(5) = 7.7, p < 0.001); Fig. 4b black bars, n = 4).

Regarding the CSD analysis, there was a prominent source under N2, qualitatively very similar in all patients (Fig. 4c overlaid maps of n = 6 patients, Supplementary Fig. S4). The detailed quantitative analysis of N2 reflected that the source was present in all layers but primarily involving the middle-upper layers with a maximum in layer III (Figs. 4b,d and 6b, n = 4). We observed greater amplitudes in supragranular versus infragranular layers (0.45 versus 0.25, grand average for normalised values, n = 4, Mann–Whitney U-test, U = 599, p = 0.031). Layer-by-layer comparison resulted in slightly significant differences revealed by Kruskal–Wallis ANOVA (χ^2^ = 16.7, p = 0.005), although post-hoc tests did not show any relevant differences between layers (Fig. 4b red bars, 4d, n = 4).

### Comparison of sSW and N2

We defined the laminar characteristics of sSW in four cases where histological reconstruction of microelectrode track was available (Pt1, 2, 3, 5), in order to compare the two types of negative potentials (i.e. the N2 and downstate of sSW) quantitatively. Supplementary Table S3 shows the total nr. of identified SWs for each patient. Examples for raw recordings of sSW and N2 are shown in Supplementary Fig. S3. Figure 5 illustrates the data of one patient (Pt1), while the results of the other three subjects can be found on Fig. 6 and Supplementary Figs. S4, S5.

Similar to our previous findings, the SWd was characterised by a negativity maximal in supragranular layers (I-III, Fig. 5 row1/a, Supplementary Fig. S4), a current source highest in the middle-upper layers (II-IV, Fig. 5 row1/b, Supplementary Fig. S4) and a MUA decrease in the middle layers (II-IV, Fig. 6 row1/c Supplementary Fig. S4), accompanied by a pronounced wideband (1–200 Hz) spectral power decrease across all cortical layers (Fig. 5 row1/d, Supplementary Fig. S4).

Figure 5e demonstrates the prominent decrease in neural firing during both SWd and N2, corresponding to neural “off” periods. The temporal association of sSW and N2 is illustrated on the overlaid LFP and CSD plots of Fig. 5f. Despite the slower progression and higher amplitudes of sSWs, the laminar characteristics of N2, described in the previous section, are highly similar to those observed by sSW. To highlight the similarities, the same N2 measures of the same patients were placed by the side of sSW data (Fig. 5, Supplementary Figs. S4, S5).

To quantify the above-mentioned similarities in LFP, CSD, MUA and TFR of N2 and sSW, we compared the amplitudes measured on individual channels. In all patients, we found a strong relationship between all parameters: across channels of the superficial negative potential in absolute and normalised amplitude (LFP; Pearson’s r = 0.92 for Pt1), in current source density (CSD; r = 0.75 for Pt1; Pt2 r = 0.76; Pt3 r = 0.88; Pt5 r = 0.878) and in MUA decrements (MUA; r = 0.79 Pt1), while the extent of spectral power decrease correlated weakly (TFR; r = 0.41 Pt1) (Fig. 5 row3, Fig. 6a). There was no significant difference in amplitude between N2 and sSW downstate in either cortical layer, both in terms of field potentials and current sources (two-sample t-tests: layer I-II p = 0.31, layer III p = 0.48, layer IV p = 0.12, layer V p = 0.09, layer VI p = 0.31; bootstrap estimation statistics for each cortical layers) (Fig. 6b,c, n = 4). Maximal amplitudes were measured in supragranular cortical layers (field potential: layer I-II, current source: layer III) for both potentials (Fig. 6b, Supplementary Figs. S4, S5). Amplitude differences between N2 and SWS downstate are illustrated at a Gardner-Altman estimation plot (Fig. 6c). Results of Bayesian Pearson correlation indicated very strong evidence (BF = 86.48) in favour of a positive correlation between N2 and SW downstate amplitude (r = 0.77, median = 0.71, 95% CI [0.39, 0.91]) in layer III. Regarding CSDs, there were strong and extreme evidences of correlation between N2 and SW downstate source amplitude in layerI-II (r = 0.76, 95% CI [0.44, 0.89], BF = 465), layer III (r = 0.68, 95% CI [0.23, 0.86], BF = 15.5), layer IV (r = 0.87, 95% CI [0.49, 0.96], BF = 143) and layer V (r = 0.86, 95% [0.62, 0.95], BF = 6290), suggesting similarities in their laminar distribution (Supplementary Table S4).

## Discussion

In our study, we used intracortical LMEs to record CCEPs in seven epileptic patients. We have described the electrophysiological changes related to various CCEP components on the level of LFP, CSD, multi- and single unit activity. Additionally, we have described the similarities between downstate of intrinsic sleep SW and the N2 component of CCEP, supporting our hypothesis that SW-like phenomena can be elicited also in the awake state.

### Different CCEP components

We were able to identify qualitatively similar CCEP components in all patients, divided into three categories based on the order of appearance, as early: P1, N1, middle: P2, N2 and late: P3 (Fig. 2a). This separation was made based on the differences of the underlying mechanisms discussed later. We could not determine clear cutoff latency thresholds between these categories, although the average latency for N1 was around 20 ms, while for the middle peaks (P2 and N2) it was around 60 ms and 150 ms respectively, and for the late peak (P3) it was 375 ms (rounded values). Latency appeared to be stable throughout the layers (Fig. 2d/2) indicating separate cortical processes during these phases (Fig. 2d/2).

We found a heterogeneity in the appearance of the components across stimulated electrode pairs even within the same patients, as described in previous studies^45^. Early components could be evoked from nearby stimulation sites, most reliably within 1 cm (Fig. 2d/5), while middle and late components had less distance dependency. The reason behind this might be that N1 is thought to be related to cortico-cortical projections within the functional networks, so its latency and amplitude might correlate with the distance and number of tracts between response and stimulation sites due to extensive short-range connections^12,13^.

On the contrary, the most often emerging component was N2, showing no distance dependence. Although not significant, the rate of N2 peaks was higher compared to other peaks’ in case of all examined distances. This corresponds to the finding that N2 reflects the cross-network propagation of the activation^13^, transmitted to both subcortical and functionally weakly connected sites, hence engaging a spatially more diffused cortical area, captured also with a higher chance by the implanted electrodes.

Interestingly, P2 and P3 components were identified in lower rates, similar to N1 peak, while in terms of stimulation distance appeared to lie in-between N1 and N2. P2 was predominantly elicited from within 1 cm but appeared more often than N1 at further stimulation sites. Conversely, P3 occurred at higher rates when stimulating from 3 cm, but less often than N2. Additionally, the average rate of P3 at nearby stimulation sites was similar to that of N1 peak. Although statistically not confirmed, these observations raise the possibility that P2 and P3 are generated by the weighted contribution of N1 and N2-related processes, respectively (Fig. 2d/5).

Whether stimulation occurred within or outside of the SOZ, this had no significant impact on the appearance rate of CCEP components, although the rate of N1 and P2 components seemed to be higher within the SOZ. There is evidence that the epileptogenic network is characterised by more accentuated short-distance connections and increased excitability, reflected also by higher N1 amplitudes recorded in the epileptogenic zone, while these features might also contribute to the higher rate of its appearance^9^. Nonetheless, the insignificant result might arise from the low sample size examined in our analysis, therefore it should be further investigated in a larger dataset.

### Laminar organisation of CCEP

We found a discrepancy between postsynaptic processes (LFP and CSD) and unit firing (MUA and SUA). Most of the CSD components were found in the superficial layers (I-III) while the MUA involved mostly the deep layers shifting upwards to the middle layers.

The early components were associated with an alternating sink-source pattern in superficial layers (I-III) starting with a layer III sink under P1 followed by a layer I-II sink during N1. There was also a low amplitude early sink in layer V during P1, as the only deep layer CSD component throughout the whole CCEP pattern. The early MUA response showed a huge increase mostly but not exclusively in the deep layers and wide-band spectral power with maxima in the infragranular layers (Fig. 2c) indicating strong excitation during this phase. Furthermore, the most prominent increase in SUA relative to baseline was found also in lower-middle layers.

Layer V neurons have extended dendritic arborization spanning across multiple layers and a widespread projection system, involving both subcortical, intra- and interlaminar connections^46^. Therefore, these cells, especially large pyramidal neurons with tufted dendritic arborization in layers I/II, are suitable drivers of stimulation-elicited responses, depolarized either through their dendritic trees or by traversing axons. In the latter case, the impulse conducted by the axons can travel both orthodromically, leading to trans-synaptic neural activation and antidromically, however backpropagation is thought to contribute to stimulation-evoked cortical responses to a lesser degree^1,38,47^. Hence, P1 most likely corresponds to suprathreshold depolarization of layer V cells.

Contrary to P1, N1 has been suggested to reflect oligo- or polysynaptic activation^38^. Activation from layer V is transmitted to all cortical layers: cells arborize densely within deep layers, leading to sustained infragranular MUA, but they also join prominent vertical axon bundles^46^. Thus, the main direction of signal propagation is vertical, as confirmed also by a recent study analysing layer-specific microstimulations of rat visual cortex^48^, stating that stimulus delivered at the level of layers V and VI mostly propagates along the cortical column^49^. Layer V cells most densely innervate layer I/II by feedback projections, both directly and by long-range connections via layer III/IV excitatory neurons, presumably resulting in a superficial sink associated with N1.

In contrast to the early ones, middle components showed surface positive (P2) and surface negative (N2) waves that were accompanied by a superficial (layer I-II) and a middle layer (III) source respectively, and were associated with decreased MUA, SUA and TFR. The neural inhibition observed here might be explained by both local and long-range inhibitory influences. Several animal studies have shown that while layers II/III contain large recurrent excitatory circuits that mediate the selective amplification of incoming sensory information, these layers also endure higher inhibitory control compared to the infragranular domain^49^. A recent study, describing the effect of intracortical microstimulation on spiking activity in awake macaque monkeys, concluded that stimulation results in inhibition of spiking, with or without preceding excitatory response^47^. In this study the authors found, that contrary to the excitation, the inhibitory responses were independent of stimulation distance (up to 4.5 mm) and appeared 5–100 ms following the stimulus. They concluded that inhibition with no excitatory response beforehand, might be caused by the activation of long horizontal fibres with stronger feedforward connections to inhibitory neurons than to principal cells, especially in case of layer II/III cells. In turn, if an excitatory response is evoked, it also activates feedback inhibitory circuits. Our observation that P2 is more likely to be elicited from closer stimulation sites (1–2 cm) supports that the decreased neural activity and superficial source during P2 is provoked by feedforward/feedback inhibition enacted by local interneurons. Given the time range (5–100 ms) of spiking suppression of individual neurons following microstimulation^47^, this mechanism might contribute to the emergence of both P2 and N2. However, based on previous literature on CCEPs, N2 component is more likely to be, at least partially, of subcortical origin^13,50^.

The P3 component identified in our data appears as a rebound following N2, with an upper-middle sink and high MUA and SUA increase. This pattern of activation is highly reminiscent of sleep SWs, pointing to a potential involvement of thalamocortical pathways (see later in details).

### N2 is qualitatively similar to local cortical downstate of sSW

Based on our findings, the intracortical laminar profile of sSW downstate and N2 component of CCEP are similar to each other. The cortical generators of the negative phase of sSW and the N2 were localised to the supragranular layers (I-III), with maximal amplitudes in layer II (Figs. 5, 6b, Supplementary Figs. S4, S5). LFP changes during N2 and sSW both demonstrated a negative potential in the upper layers followed by a positive potential in the same layers during P3. Biphasic CSD responses (source in N2, sink in P3) primarily localised to the middle-upper layers (II-IV) representing the activation of supragranular portion of the cortex after cortical stimulation. Both potentials exhibited wideband TFR decreases and increases during the N2 and P3 responses, respectively. These changes were all similar to sSW (Fig. 5, Supplementary Fig. S4). A typical cortical downstate is characterised by neuronal silence, outward currents from neurons, in the upper-middle layers, and wideband power decreases^16,51^. According to these criteria we can speculate that N2 represents a single downstate of a SW cycle. The rebound excitation following N2, the P3 shows alternate properties like excessive neuronal firing, inward currents on the CSD and spectral power increase, that are characteristic to the laminar phenomena of cortical upstates^16,23,52,53^. Therefore, we think that these components together represent one complete sleep slow cycle.

There is evidence from animal^34^ and human cortical electrical^31^ and transcranial magnetic stimulation^32,33^ studies that SWs can be triggered during NREM sleep. SWs evoked by microstimulation of rat cortex showed remarkable similarity to sSWs^34^. Likewise, it has been found that TMS evoked SWs in human during NREM sleep have similar features and spreading patterns over the scalp to spontaneous ones^32^. Additionally, both spontaneous and sensory-evoked K-complexes were shown to share similar intracortical disfacilitation as spontaneous SWs in epileptic patients implanted with laminar microelectrodes^37^.

Spontaneous local cortical sleep-like phenomena were observed on cortical regional or columnar level in awake state related to sleep pressure in rats^54^ and human participants^55^, but also linked to attentional lapses in not sleep-deprived human subject^56,57^, supporting that local sleep phenomena might co-occur with awake brain functioning.

In turn, there are few and partially contradicting results on whether and in what form SWs can be elicited in the awake state^32,58^. Massimini et al. ^32^ state that they could not evoke SWs in awake. The difference between these and our results might arise from the non-invasive nature of the recording technique since EEG combined with TMS cannot record the precise local effects of the stimulation. Pigorini et al.^58^ were able to trigger SWs in a more activated, low amplitude, ‘wakefulness-like’ phase of sleep, and in few cases, also in the awake state, when evoked potentials were recorded from the vicinity of stimulated electrodes. Nevertheless, they claim that CCEPs triggered in awake generally lack the suppression of high frequencies characteristic to SWs. The discrepancy between this and our result might arise from the more localised nature of this phenomena during wakefulness, captured only by contacts close to stimulation and microelectrodes.

Despite these similarities in laminar distribution of cortical generators, sSWs have higher amplitudes and a slower characteristic than the N2-P3 cycle, although it has to be noted that while the downstate-upstate cycle is indeed slower, the duration of downstate might be comparable to N2 (Fig. 5f) Regarding the amplitude difference, this is in line with previous findings that both sensory^59^ and electrically evoked responses are of larger magnitude during NREM sleep, than in awake state. Considering differential durations, sleep SWs typically consist of a 0.3–0.4 Hz cycle with some variance up to 1 Hz in animals^60^. Despite this frequency interval for SW, which is broader in humans (0.5–2 Hz^61^), the cortical pattern of short and long cycles were found to be identical^16^. Additionally, there is evidence^62^ that downstate-upstate durations can be variable even within an oscillatory cycle. Additionally, both animal^54^ and human^55^ studies showed that extensive use of certain brain areas results not only in increased SW frequency during sleep but also in greater theta power density when awake in the same cortical areas^63^. In the work of Vyazovskiy^54^, these theta frequency waves, recorded in sleep-deprived, but awake and behaving rats, were related with neuronal OFF periods. It might be possible that the same neural mechanisms manifest with slightly different durations depending on vigilance state. Based on these results, we think that the frequency itself is not characteristic to this type of cortical oscillation, but the laminar distribution reflects more the similarities of the underlying neural processes. The faster frequency of the N2 component of the CCEP may reflect more localised cortical participation after SPES than the widespread involvement in sleep. Similar phenomena can be observed studying cortical slowing associated with pathological processes, where the frequency rather reflects the severity and the volume of the cortical malfunction than the etiology^64^.

### Hypothesised intracortical activation sequence during CCEP

**Figure 7:**
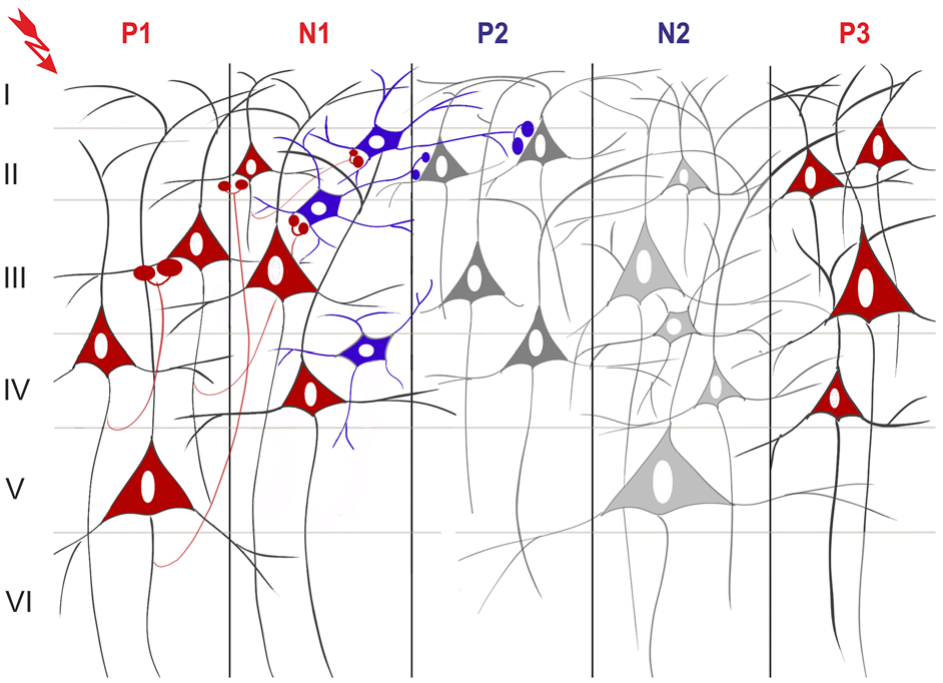
Hypothetic intracortical activation sequence of evoked potentials. Based on laminar profile of CCEPs and results of single-unit analysis (SUA), there is a direct activation of deep layer pyramidal cells induced by electrical stimulus (P1), which is followed by apical dendritic excitation (N1) either by short range recurrent connections, or through activation of layer III-IV excitatory neurons. A feedback inhibitory mechanism or contribution of upper layer interneurons can explain the hyperpolarization and SUA decrease in supragranular layers during P2 component. The N2 and P3 components represent an evoked sleep slow cycle, and are characterised by a wide middle layer source with a marked decrease in SUA, suggesting disfacilitation of a downstate (N2), and a subsequent upstate (P3) with excitation of the neurons both in middle and especially in upper cortical layers. The transition between P2 and N2 is unclear, but may involve thalamic contribution. Colored neurons represent neuronal firing (red: excitatory, blue: inhibitory), colored buttons represent synaptic activation. Gray shaded neurons represent inactive or inhibited firing.

Based on our results, we created a hypothetic intracortical activation sequence to model the local cortical processes during SPES evoked potentials (Fig. 7). The early excitation depolarizes layer V pyramidal neurons (P1) that contributes to a local feed-forward excitation (N1) delivered to layers I-II, which disengages local inhibitory processes, contributing to active inhibition in P2. After this, a sleep SW-like cycle arises: a downstate representing all layer disfacilitation^20^ (N2), followed by an upstate (P3). The transition mechanism between local neuronal processes (early components and P2) and evoked SW (N2 and P3) is not known. According to our hypothesis, the cortical inhibition during P2 might propagate to the thalamus and trigger SW^65^.

## Limitations

We were able to perform histological verification of recorded cortical laminae in only four cases. Our low number of samples has an internal limitation on the conclusions, so detailed comparison of evoked and spontaneous LFP activity would need further studies. To counteract this limitation, we utilised bootstrap based estimation ~ and Bayesian statistics that verified the non-significant difference between the two processes. The limited number of the sample is due to the technical challenge of this type of recordings in humans. The seven patients were selected from twenty patients with laminar electrodes. Eight patient’s data was excluded due to bad data quality, and further five cases missed CCEP experiment.

Additionally, although we have not detected any structural damage in the LME containing blocks with available histology, the pathologic reorganisation of the local cortical network due to the proximity of the hypothesised SOZ cannot be excluded.

Another limitation of our study is that we were able to apply stimulation only on electrodes positioned superficially on the cortex. It is possible that the delivery of microstimulations to various layers of the cortex might yield different results regarding laminar pattern and distance dependence of evoked potentials, the clarification of which would be highly interesting. However, we think that our data might still be relevant from a clinical point of view, considering that diagnostic stimulatory protocols in this field are similar to that applied in our cohort.

## Conclusion

In sum, we have described the emerging intracortical activation pattern elicited by SPES and sought to build a hypothetical model capturing the local cortical propagation pattern.

Additionally, based on our results (even if they are mostly qualitative) and the current literature about local SW-like processes we think that the comparison of stimulation-evoked and sleep phenomena provides a valuable ground for developing a hypothesis regarding the sleep—wakefulness relations of fundamental working mechanisms of the brain. Our findings might contribute to the better understanding of local cortical processes during cortical stimulation, with the final aim of helping to improve neuromodulatory techniques.

## Supplementary Information

Supplementary Tables.

Supplementary Figures.

## Data Availability

The data that support the findings of this study are available on request from the corresponding author. The data are not publicly available due to privacy or ethical restrictions. The custom-made algorithms used for data acquisition and analysis are freely available on request from the corresponding author.

## Abbreviations

CCEP: Cortico-cortical evoked potential
CSD: Current source density
CT: Computed tomography
EcoG: Electro-corticography
EEG: Electroencephalogram
ERSP: Event-related spectral perturbation
LFP: Local field potential
LME: Laminar microelectrodes
MRI: Magnetic resonance imaging
MUA: Multiple unit activity
SPES: Single-pulse electric stimulation
SUA: Single-unit activity
SW: Slow wave
sSW: Spontaneous SW
SWd: SW downstate
TFR: Time–frequency response
TMS: Transcranial magnetic stimulation

## References

[1] Entz, L. *et al.* Evoked effective connectivity of the human neocortex. *Hum. Brain Mapp.* **35**, 5736–5753. 10.1002/hbm.22581 (2014). 25044884 10.1002/hbm.22581 PMC4797947

[2] Valentin, A. *et al.* Single pulse electrical stimulation for identification of structural abnormalities and prediction of seizure outcome after epilepsy surgery: A prospective study. *Lancet Neurol.* **4**, 718–726. 10.1016/S1474-4422(05)70200-3 (2005). 16239178 10.1016/S1474-4422(05)70200-3

[3] Valentin, A. *et al.* Single-pulse electrical stimulation identifies epileptogenic frontal cortex in the human brain. *Neurology* **65**, 426–435. 10.1212/01.wnl.0000171340.73078.c1 (2005). 16087908 10.1212/01.wnl.0000171340.73078.c1

[4] Alarcon, G. *et al.* In vivo neuronal firing patterns during human epileptiform discharges replicated by electrical stimulation. *Clin. Neurophysiol.* **123**, 1736–1744. 10.1016/j.clinph.2012.02.062 (2012). 22410162 10.1016/j.clinph.2012.02.062 PMC3432232

[5] Boulogne, S., Ryvlin, P. & Rheims, S. Single and paired-pulse electrical stimulation during invasive EEG recordings. *Rev. Neurol.* **172**, 174–181. 10.1016/j.neurol.2016.02.004 (2016). 26993563 10.1016/j.neurol.2016.02.004

[6] Enatsu, R. *et al.* Correlations between ictal propagation and response to electrical cortical stimulation: A cortico-cortical evoked potential study. *Epilepsy Res.* **101**, 76–87. 10.1016/j.eplepsyres.2012.03.004 (2012). 22459638 10.1016/j.eplepsyres.2012.03.004

[7] Yamao, Y. *et al.* Intraoperative brain mapping by cortico-cortical evoked potential. *Front. Hum. Neurosci.* **15**, 635453. 10.3389/fnhum.2021.635453 (2021). 33679353 10.3389/fnhum.2021.635453 PMC7930065

[8] Yu, X. *et al.* Cortico-cortical evoked potentials in children with tuberous sclerosis complex using stereo-electroencephalography. *Front. Neurol.* **10**, 1093. 10.3389/fneur.2019.01093 (2019). 31736846 10.3389/fneur.2019.01093 PMC6828959

[9] Zhao, C. *et al.* Localization of epileptogenic zone based on cortico-cortical evoked potential (CCEP): A feature extraction and graph theory approach. *Front. Neuroinform.* **13**, 31. 10.3389/fninf.2019.00031 (2019). 31068798 10.3389/fninf.2019.00031 PMC6491865

[10] Matsumoto, R. *et al.* Functional connectivity in human cortical motor system: A cortico-cortical evoked potential study. *Brain* **130**, 181–197. 10.1093/brain/awl257 (2007). 17046857 10.1093/brain/awl257

[11] Matsumoto, R. *et al.* Functional connectivity in the human language system: A cortico-cortical evoked potential study. *Brain* **127**, 2316–2330. 10.1093/brain/awh246 (2004). 15269116 10.1093/brain/awh246

[12] Kunieda, T., Yamao, Y., Kikuchi, T. & Matsumoto, R. New approach for exploring cerebral functional connectivity: Review of cortico-cortical evoked potential. *Neurol. Med. Chir.* **55**, 374–382. 10.2176/nmc.ra.2014-0388 (2015). 10.2176/nmc.ra.2014-0388 PMC4628165 25925755

[13] Veit, M. J. *et al.* Temporal order of signal propagation within and across intrinsic brain networks. *Proc. Natl. Acad. Sci. USA*10.1073/pnas.2105031118 (2021). 34819365 10.1073/pnas.2105031118 PMC8640784

[14] Terada, K. *et al.* Uneven interhemispheric connections between left and right primary sensori-motor areas. *Hum. Brain Mapp.* **33**, 14–26. 10.1002/hbm.21189 (2012). 21337473 10.1002/hbm.21189 PMC6870242

[15] Ulbert, I., Halgren, E., Heit, G. & Karmos, G. Multiple microelectrode-recording system for human intracortical applications. *J. Neurosci. Methods* **106**, 69–79. 10.1016/s0165-0270(01)00330-2 (2001). 11248342 10.1016/s0165-0270(01)00330-2

[16] Csercsa, R. *et al.* Laminar analysis of slow wave activity in humans. *Brain* **133**, 2814–2829. 10.1093/brain/awq169 (2010). 20656697 10.1093/brain/awq169 PMC3105490

[17] Ulbert, I., Heit, G., Madsen, J., Karmos, G. & Halgren, E. Laminar analysis of human neocortical interictal spike generation and propagation: Current source density and multiunit analysis in vivo. *Epilepsia* **45**(Suppl 4), 48–56. 10.1111/j.0013-9580.2004.04011.x (2004). 15281959 10.1111/j.0013-9580.2004.04011.x

[18] Fabo, D. *et al.* Properties of in vivo interictal spike generation in the human subiculum. *Brain* **131**, 485–499. 10.1093/brain/awm297 (2008). 18083752 10.1093/brain/awm297

[19] Kales, A., Rechtschaffen, A., University of California, L. A. B. I. S. & Network, N. N. I. *A Manual of Standardized Terminology, Techniques and Scoring System for Sleep Stages of Human Subjects: Allan Rechtschaffen and Anthony Kales, Editors*. (U. S. National Institute of Neurological Diseases and Blindness, Neurological Information Network, 1968).

[20] Steriade, M., Nunez, A. & Amzica, F. A novel slow (< 1 Hz) oscillation of neocortical neurons in vivo: Depolarizing and hyperpolarizing components. *J. Neurosci.* **13**, 3252–3265. 10.1523/JNEUROSCI.13-08-03252.1993 (1993). 8340806 10.1523/JNEUROSCI.13-08-03252.1993 PMC6576541

[21] Achermann, P. & Borbely, A. A. Low-frequency (< 1 Hz) oscillations in the human sleep electroencephalogram. *Neuroscience* **81**, 213–222. 10.1016/s0306-4522(97)00186-3 (1997). 9300413 10.1016/s0306-4522(97)00186-3

[22] Massimini, M., Huber, R., Ferrarelli, F., Hill, S. & Tononi, G. The sleep slow oscillation as a traveling wave. *J. Neurosci.* **24**, 6862–6870. 10.1523/jneurosci.1318-04.2004 (2004). 15295020 10.1523/JNEUROSCI.1318-04.2004 PMC6729597

[23] Sanchez-Vives, M. V. & McCormick, D. A. Cellular and network mechanisms of rhythmic recurrent activity in neocortex. *Nat. Neurosci.* **3**, 1027–1034. 10.1038/79848 (2000). 11017176 10.1038/79848

[24] Sheroziya, M. & Timofeev, I. Global intracellular slow-wave dynamics of the thalamocortical system. *J. Neurosci.* **34**, 8875–8893. 10.1523/JNEUROSCI.4460-13.2014 (2014). 24966387 10.1523/JNEUROSCI.4460-13.2014 PMC4069359

[25] Fiath, R. *et al.* Laminar analysis of the slow wave activity in the somatosensory cortex of anesthetized rats. *Eur. J. Neurosci.* **44**, 1935–1951. 10.1111/ejn.13274 (2016). 27177594 10.1111/ejn.13274

[26] Zaforas, M. *et al.* Cortical layer-specific modulation of neuronal activity after sensory deprivation due to spinal cord injury. *J. Physiol.* **599**, 4643–4669. 10.1113/JP281901 (2021). 34418097 10.1113/JP281901 PMC9292026

[27] Timofeev, I. *et al.* Spatio-temporal properties of sleep slow waves and implications for development. *Curr. Opin. Physiol.* **15**, 172–182. 10.1016/j.cophys.2020.01.007 (2020). 32455180 10.1016/j.cophys.2020.01.007 PMC7243595

[28] Amzica, F. & Steriade, M. Short- and long-range neuronal synchronization of the slow (< 1 Hz) cortical oscillation. *J. Neurophysiol.* **73**, 20–38. 10.1152/jn.1995.73.1.20 (1995). 7714565 10.1152/jn.1995.73.1.20

[29] Murphy, M. *et al.* Source modeling sleep slow waves. *Proc. Natl. Acad. Sci.* **106**, 1608–1613. 10.1073/pnas.0807933106 (2009). 19164756 10.1073/pnas.0807933106 PMC2635823

[30] James, M. K., Joseph, T. N., Cheryl, J.D.-A. & Ping, T. Local sleep. *Sleep Med. Rev.* **43**, 14–21. 10.1016/j.smrv.2018.10.001 (2019). 30502497 10.1016/j.smrv.2018.10.001 PMC6351167

[31] Marshall, L., Helgadottir, H., Molle, M. & Born, J. Boosting slow oscillations during sleep potentiates memory. *Nature* **444**, 610–613. 10.1038/nature05278 (2006). 17086200 10.1038/nature05278

[32] Massimini, M. *et al.* Triggering sleep slow waves by transcranial magnetic stimulation. *Proc. Natl. Acad. Sci. USA* **104**, 8496–8501. 10.1073/pnas.0702495104 (2007). 17483481 10.1073/pnas.0702495104 PMC1895978

[33] Massimini, M., Tononi, G. & Huber, R. Slow waves, synaptic plasticity and information processing: Insights from transcranial magnetic stimulation and high-density EEG experiments. *Eur. J. Neurosci.* **29**, 1761–1770. 10.1111/j.1460-9568.2009.06720.x (2009). 19473231 10.1111/j.1460-9568.2009.06720.x PMC2776746

[34] Vyazovskiy, V. V., Faraguna, U., Cirelli, C. & Tononi, G. Triggering slow waves during NREM sleep in the rat by intracortical electrical stimulation: Effects of sleep/wake history and background activity. *J. Neurophysiol.* **101**, 1921–1931. 10.1152/jn.91157.2008 (2009). 19164101 10.1152/jn.91157.2008 PMC2695630

[35] Frauscher, B. *et al.* Facilitation of epileptic activity during sleep is mediated by high amplitude slow waves. *Brain* **138**, 1629–1641. 10.1093/brain/awv073 (2015). 25792528 10.1093/brain/awv073 PMC4614129

[36] Ujma, P. P., Halasz, P., Kelemen, A., Fabo, D. & Eross, L. Epileptic interictal discharges are more frequent during NREM slow wave downstates. *Neurosci. Lett.* **658**, 37–42. 10.1016/j.neulet.2017.08.020 (2017). 28811195 10.1016/j.neulet.2017.08.020

[37] Cash, S. S. *et al.* The human K-complex represents an isolated cortical down-state. *Science* **324**, 1084–1087. 10.1126/science.1169626 (2009). 19461004 10.1126/science.1169626 PMC3715654

[38] Keller, C. J. *et al.* Corticocortical evoked potentials reveal projectors and integrators in human brain networks. *J. Neurosci.* **34**, 9152–9163. 10.1523/JNEUROSCI.4289-13.2014 (2014). 24990935 10.1523/JNEUROSCI.4289-13.2014 PMC4078089

[39] Ulbert, I. *et al.* In vivo laminar electrophysiology co-registered with histology in the hippocampus of patients with temporal lobe epilepsy. *Exp. Neurol.* **187**, 310–318. 10.1016/j.expneurol.2003.12.003 (2004). 15144857 10.1016/j.expneurol.2003.12.003

[40] Turner, D. A., Li, X. G., Pyapali, G. K., Ylinen, A. & Buzsaki, G. Morphometric and electrical properties of reconstructed hippocampal CA3 neurons recorded in vivo. *J. Comp. Neurol.* **356**, 580–594. 10.1002/cne.903560408 (1995). 7560268 10.1002/cne.903560408

[41] Wittner, L., Henze, D. A., Zaborszky, L. & Buzsaki, G. Hippocampal CA3 pyramidal cells selectively innervate aspiny interneurons. *Eur. J. Neurosci.* **24**, 1286–1298. 10.1111/j.1460-9568.2006.04992.x (2006). 16987216 10.1111/j.1460-9568.2006.04992.x

[42] Delorme, A. & Makeig, S. EEGLAB: An open source toolbox for analysis of single-trial EEG dynamics including independent component analysis. *J. Neurosci. Methods* **134**, 9–21. 10.1016/j.jneumeth.2003.10.009 (2004). 15102499 10.1016/j.jneumeth.2003.10.009

[43] Nicholson, C. & Freeman, J. A. Theory of current source-density analysis and determination of conductivity tensor for anuran cerebellum. *J. Neurophysiol.* **38**, 356–368. 10.1152/jn.1975.38.2.356 (1975). 805215 10.1152/jn.1975.38.2.356

[44] Ho, J., Tumkaya, T., Aryal, S., Choi, H. & Claridge-Chang, A. Moving beyond P values: Data analysis with estimation graphics. *Nat. Methods* **16**, 565–566. 10.1038/s41592-019-0470-3 (2019). 31217592 10.1038/s41592-019-0470-3

[45] Keller, C. J. *et al.* Mapping human brain networks with cortico-cortical evoked potentials. *Philos. Trans. R. Soc. Lond. B Biol. Sci.*10.1098/rstb.2013.0528 (2014). 25180306 10.1098/rstb.2013.0528 PMC4150303

[46] Thomson, A. M. & Bannister, A. P. Interlaminar connections in the neocortex. *Cereb. Cortex* **13**, 5–14. 10.1093/cercor/13.1.5 (2003). 12466210 10.1093/cercor/13.1.5

[47] Yun, R., Mishler, J. H., Perlmutter, S. I., Rao, R. P. N. & Fetz, E. E. Responses of cortical neurons to intracortical microstimulation in awake primates. *eNeuro*10.1523/ENEURO.0336-22.2023 (2023). 37037604 10.1523/ENEURO.0336-22.2023 PMC10135083

[48] Allison-Walker, T., Hagan, M. A., Price, N. S. C. & Wong, Y. T. Microstimulation-evoked neural responses in visual cortex are depth dependent. *Brain Stimul.* **14**, 741–750. 10.1016/j.brs.2021.04.020 (2021). 33975054 10.1016/j.brs.2021.04.020

[49] D’Souza, R. D. & Burkhalter, A. A laminar organization for selective cortico-cortical communication. *Front. Neuroanat.* **11**, 71. 10.3389/fnana.2017.00071 (2017). 28878631 10.3389/fnana.2017.00071 PMC5572236

[50] Matsumoto, R., Kunieda, T. & Nair, D. Single pulse electrical stimulation to probe functional and pathological connectivity in epilepsy. *Seizure* **44**, 27–36. 10.1016/j.seizure.2016.11.003 (2017). 27939100 10.1016/j.seizure.2016.11.003 PMC5291825

[51] Neske, G. T. The slow oscillation in cortical and thalamic networks: Mechanisms and functions. *Front. Neural Circuits* **9**, 88. 10.3389/fncir.2015.00088 (2015). 26834569 10.3389/fncir.2015.00088 PMC4712264

[52] Chauvette, S., Volgushev, M. & Timofeev, I. Origin of active states in local neocortical networks during slow sleep oscillation. *Cereb. Cortex* **20**, 2660–2674. 10.1093/cercor/bhq009 (2010). 20200108 10.1093/cercor/bhq009 PMC2951844

[53] Sakata, S. & Harris, K. D. Laminar structure of spontaneous and sensory-evoked population activity in auditory cortex. *Neuron* **64**, 404–418. 10.1016/j.neuron.2009.09.020 (2009). 19914188 10.1016/j.neuron.2009.09.020 PMC2778614

[54] Vyazovskiy, V. V. *et al.* Local sleep in awake rats. *Nature* **472**, 443–447. 10.1038/nature10009 (2011). 21525926 10.1038/nature10009 PMC3085007

[55] Hung, C. S. *et al.* Local experience-dependent changes in the wake EEG after prolonged wakefulness. *Sleep* **36**, 59–72. 10.5665/sleep.2302 (2013). 23288972 10.5665/sleep.2302 PMC3524543

[56] Andrillon, T., Burns, A., Mackay, T., Windt, J. & Tsuchiya, N. Predicting lapses of attention with sleep-like slow waves. *Nat. Commun.* **12**, 3657. 10.1038/s41467-021-23890-7 (2021). 34188023 10.1038/s41467-021-23890-7 PMC8241869

[57] Quercia, A., Zappasodi, F., Committeri, G. & Ferrara, M. Local use-dependent sleep in wakefulness links performance errors to learning. *Front. Hum. Neurosci.* **12**, 122. 10.3389/fnhum.2018.00122 (2018). 29666574 10.3389/fnhum.2018.00122 PMC5891895

[58] Pigorini, A. *et al.* Bistability breaks-off deterministic responses to intracortical stimulation during non-REM sleep. *Neuroimage* **112**, 105–113. 10.1016/j.neuroimage.2015.02.056 (2015). 25747918 10.1016/j.neuroimage.2015.02.056

[59] Rector, D. M., Topchiy, I. A., Carter, K. M. & Rojas, M. J. Local functional state differences between rat cortical columns. *Brain Res.* **1047**, 45–55. 10.1016/j.brainres.2005.04.002 (2005). 15882842 10.1016/j.brainres.2005.04.002

[60] Steriade, M., Nunez, A. & Amzica, F. Intracellular analysis of relations between the slow (< 1 Hz) neocortical oscillation and other sleep rhythms of the electroencephalogram. *J. Neurosci.* **13**, 3266–3283. 10.1523/jneurosci.13-08-03266.1993 (1993). 8340807 10.1523/JNEUROSCI.13-08-03266.1993 PMC6576520

[61] Iber, C., American Academy of Sleep, M. *The AASM Manual for the Scoring of Sleep and Associated Events: Rules, Terminology and Technical Specifications* (American Academy of Sleep Medicine, 2007).

[62] Torao-Angosto, M., Manasanch, A., Mattia, M. & Sanchez-Vives, M. V. Up and down states during slow oscillations in slow-wave sleep and different levels of anesthesia. *Front. Syst. Neurosci.* **15**, 609645. 10.3389/fnsys.2021.609645 (2021). 33633546 10.3389/fnsys.2021.609645 PMC7900541

[63] Andrillon, T. & Oudiette, D. What is sleep exactly? Global and local modulations of sleep oscillations all around the clock. *Neurosci. Biobehav. Rev.* **155**, 105465. 10.1016/j.neubiorev.2023.105465 (2023). 37972882 10.1016/j.neubiorev.2023.105465

[64] Britton, J. W. *et al.**Electroencephalography (EEG): An Introductory Text and Atlas of Normal and Abnormal Findings in Adults, Children, and Infants* (eds St. Louis, E. K. & Frey, L. C.) (2016). 27748095

[65] Dossi, R. C., Nunez, A. & Steriade, M. Electrophysiology of a slow (0.5–4 Hz) intrinsic oscillation of cat thalamocortical neurones in vivo. *J. Physiol.* **447**, 215–234. 10.1113/jphysiol.1992.sp018999 (1992). 1593448 10.1113/jphysiol.1992.sp018999 PMC1176033

