# Author Correction: Intracortical mechanisms of single pulse electrical stimulation (SPES) evoked excitations and inhibitions in humans

**DOI:** 10.1038/s41598-024-76651-z

**Published:** 2024-10-18

**Authors:** Boglárka Hajnal, Johanna Petra Szabó, Emília Tóth, Corey J. Keller, Lucia Wittner, Ashesh D. Mehta, Loránd Erőss, István Ulbert, Dániel Fabó, László Entz

**Affiliations:** 1 Epilepsy Center, Clinic for Neurosurgery and Neurointervention, Semmelweis University, Budapest, 1145 Hungary; 2 János Szentágothai Neurosciences Program, Semmelweis University School of PhD Studies, Budapest, 1083 Hungary; 3 Department of Functional Neurosurgery, Clinic for Neurosurgery and Neurointervention, Semmelweis University, Budapest, 1145 Hungary; 4 Epilepsy and Cognitive Neurophysiology Laboratory, University of Alabama at Birmingham, Birmingham, AL 35294 USA; 5 Department of Neuroscience, Albert Einstein College of Medicine, Bronx, NY 10461 USA; 6 Department of Neurosurgery, Hofstra North Shore LIJ School of Medicine and Feinstein Institute of Medical Research, 300 Community Drive, Manhasset, NY 11030 USA; 7 Department of Neuroscience, Psychiatry and Behavioral Sciences, Stanford University, Palo Alto, CA 94304 USA; 8 Institute of Cognitive Neuroscience and Psychology, Research Centre for Natural Sciences, HUN-REN, Budapest, 1117 Hungary; 9 Department of Information Technology and Bionics, Péter Pázmány Catholic University, Budapest, 1083 Hungary; 10 Lendület Laboratory of Systems Neuroscience, HUN-REN Institute of Experimental Medicine, Budapest, 1083 Hungary

Correction to: *Scientific Reports *10.1038/s41598-024-62433-0, published online 14 June 2024

The original version of this Article contained an error in the Acknowledgements section.

“The authors thank Tibor Nanasi for help with data analysis, and László Papp (Neuronelektród Ltd, Budapest, Hungary) for manufacturing the laminar microelectrodes. The authors are enormously indebted to the patients that participated in this study, and all the staff in the epilepsy monitoring unit, and operating room of Clinic for Neurosurgery and Neurointervention, Semmelweis University. This work was supported by National Research, Development and Innovation Office, Hungary (KTIA-NAP 13-1-2013 and 2017-1.2.1-NKP-00002 to DF, LoE), Hungarian National Research Fund, Hungary (OTKA K119443 to LW), National Institutes of Health, USA (NIH/NINDS U01 NS098976-01 to AM; 2R01NS062092-07 to DF), National Institutes of Mental Health, USA (NIMH R21 MH114166-01 to AM).”

now reads:

“The authors thank Tibor Nanasi for help with data analysis, and László Papp (Neuronelektród Ltd, Budapest, Hungary) for manufacturing the laminar microelectrodes. The authors are enormously indebted to the patients that participated in this study, and all the staff in the epilepsy monitoring unit, and operating room of Clinic for Neurosurgery and Neurointervention, Semmelweis University. This work was supported by National Research, Development and Innovation Office, Hungary (KTIA-NAP 13-1-2013 and 2017-1.2.1-NKP-00002 to DF, LoE), Hungarian National Research Fund, Hungary (OTKA K137889 to LW), National Institutes of Health, USA (NIH/NINDS U01 NS098976-01 to AM; 2R01NS062092-07 to DF), National Institutes of Mental Health, USA (NIMH R21 MH114166-01 to AM).”

The original Article has been corrected.

