# Supplementary material for: Intracortical mechanisms of single pulse electrical stimulation (SPES) evoked excitations and inhibitions in humans
Source: Sci Rep. 2024 Jun 14;14:13784. doi: 10.1038/s41598-024-62433-0 (PMC11178858; doi:10.1038/s41598-024-62433-0)
Supplement: Supplementary file 2 — Supplementary Figures. [file 41598_2024_62433_MOESM2_ESM.docx]

**Supplementary material**

**Supplementary Figure S1**. **LFP and CSD pattern associated with early CCEP components**

a) LFP pattern associated with the CCEPs of 2 patients, focusing on the presentation of early components (P1, N1, P2). b) CSD maps corresponding to events presented in a). Note that the CSD map of Pt6 contains a high amount of artifacts due to low quality recording, because CSD is especially sensitive to low signal-to-noise ratio.

**
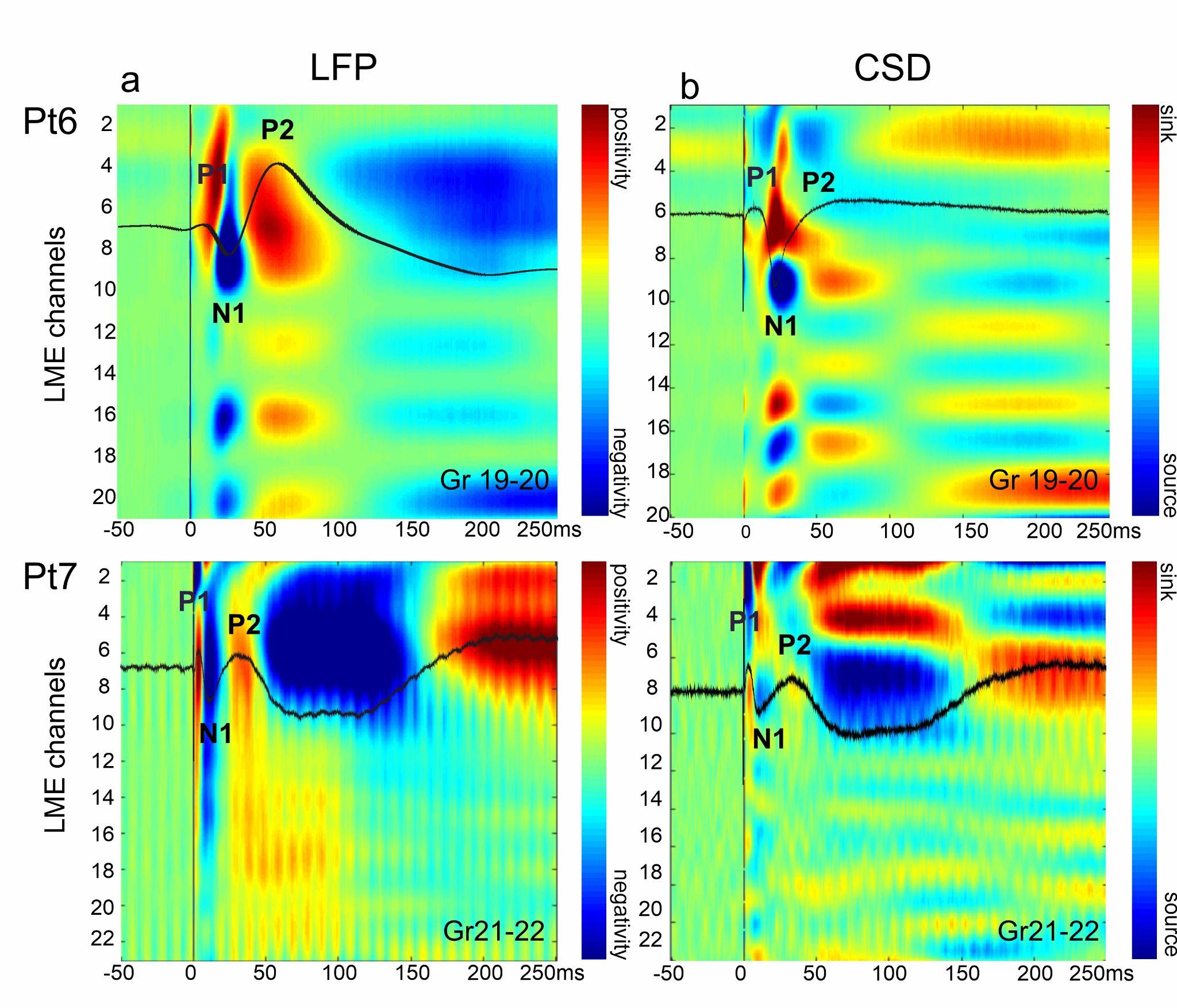
**

**Supplementary Figure S2. CSD pattern of CCEPs corresponding to preferred and non-preferred stimulation sites for each patient**

The first column shows the 3D reconstruction of the implanted electrodes (red dots) with marked preferred (blue dots) and non-preferred (yellow-dots) stimulation sites. Data derives from laminar multi-electrodes (green dots). Second column shows the LFP (from channel with maximal amplitude) and CSD pattern associated with preferred sites. The third column shows several LFP curves (from channel with maximal amplitude) derived from sites marked with yellow dots on reconstruction images and CSD maps from a selected non-preferred site (stimulating channel-pair indicated above LFP curves and in the left lower corner of CSD maps). Note that patterns of preferred and non-preferred locations are qualitatively very similar, although the latter contain more noise. All data are plotted in a window of -250 and 500 ms around stimulation.

**
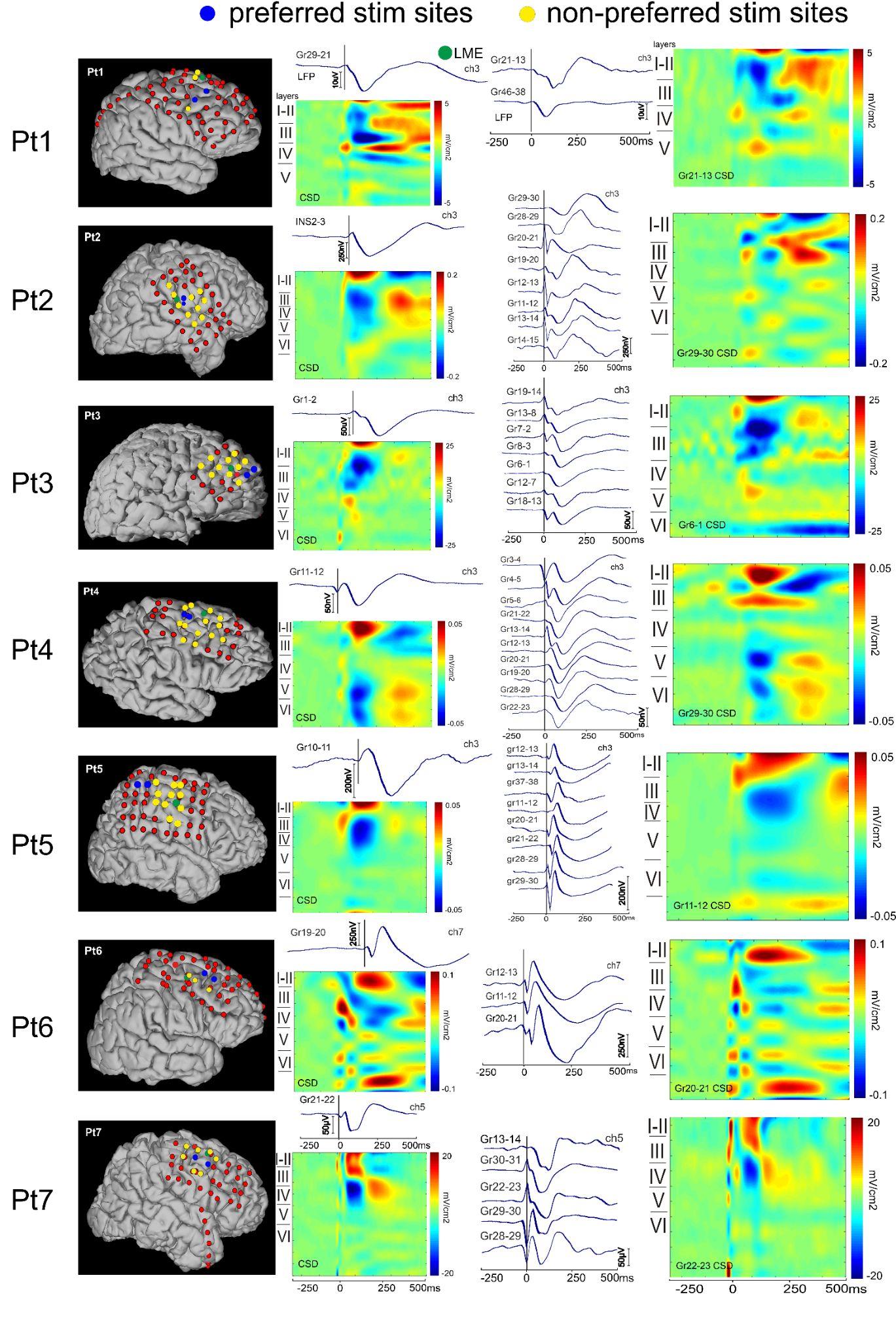
**

**Supplementary Figure S3. Raw recordings of slow wave sleep (SWS) and single-pulse electric stimulation (SPES) for two example patients**

**a)** Raw recording of N3 stage sleep with SWs in two example patients. Inset shows the average of detected SWs in these patients, on a selected channel, aligned to downstate peak. The inset in case of Pt1 also shows MUA activity (top curve) associated with the SW.

**b)** Raw recording of SPES session in two example patients. Note that in Pt1 a high pass filtered recording is shown to demonstrate the changes in MUA activity after stimulation. Black stars show the time of stimulation. Inset shows the average of detected CCEPs obtained from the stimulation of one channel-pair, on a selected channel, aligned to stimulation. Inset in case of Pt3 illustrates the processing steps of CCEP signal: zoomed-in raw recording (top curve), average raw CCEP (middle curve), average filtered CCEP (bottom curve). Inset in case of Pt1 shows MUA activity (top curve) associated with the CCEP signal. Note the different amplitude scales in SWS compared to SPES recordings.

**
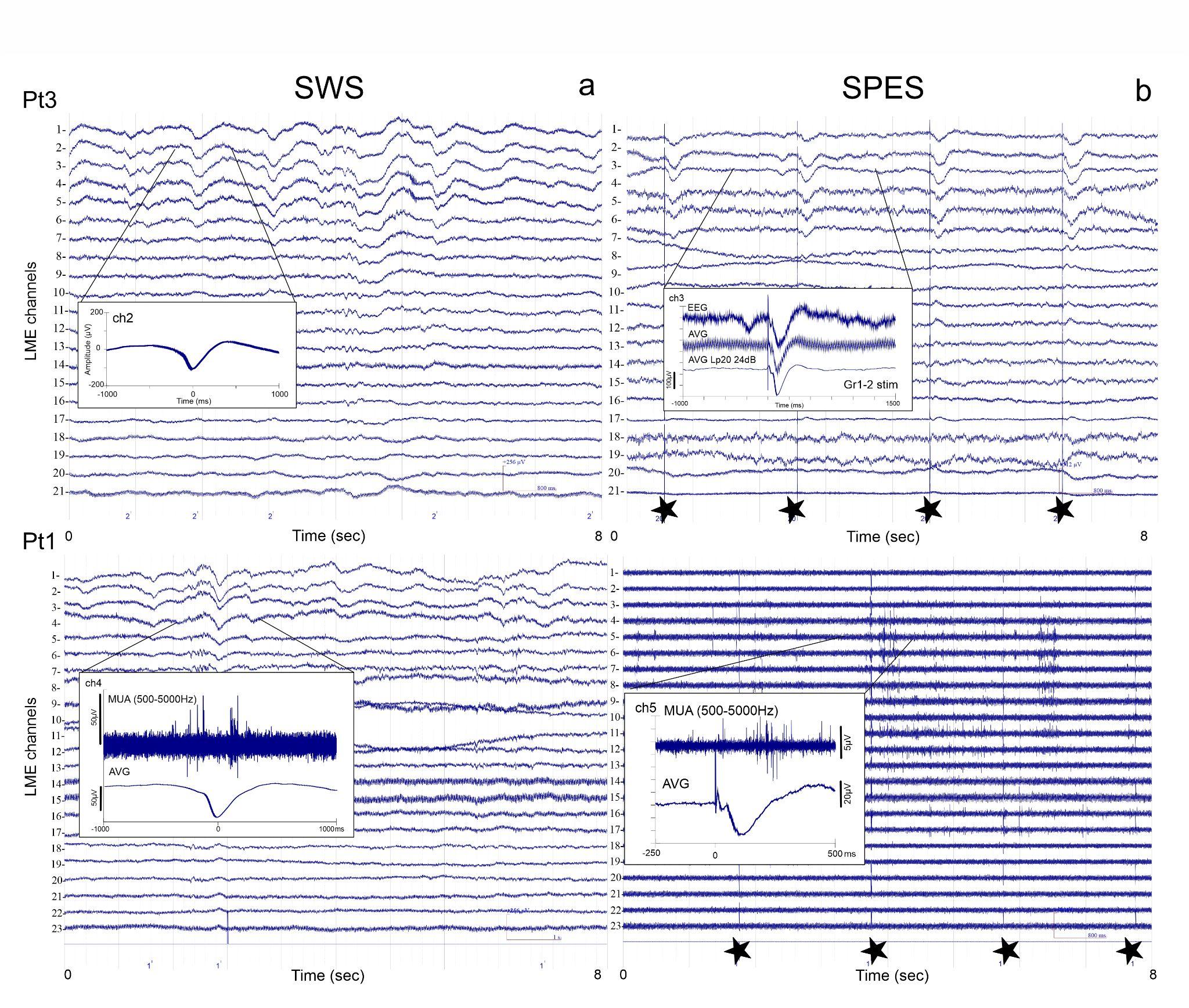
**

**Supplementary Figure S4. Comparison of intra-cortical laminar profile of N2 and spontaneous slow wave downstate (SWd) based on distribution of local field potential (LFP), current source density (CSD), multiunit activity (MUA) and time-frequency response (TFR).**

**a)** Color maps illustrate the laminar pattern of CCEPs and slow waves in three patients. The maximal negativity, measured both during the N2 component of CCEP and SWd, is localized to upper cortical layers (**a) first row**). In the same time window, current sources can be detected in supragranular layers, both during N2 and SWd (**a) second row**), suggesting similar local cortical network processes in their background. Red arrows show the time of electrical stimulus. In Pt2 MUA and TFR show similar pattern in time window of both N2 and SWd (**a) third row**)

**b)** Steps of CSD analysis of evoked potentials in Pt2 (note the originally bad channels 14 and 15 are interpolated with a smoothing hamming filter).


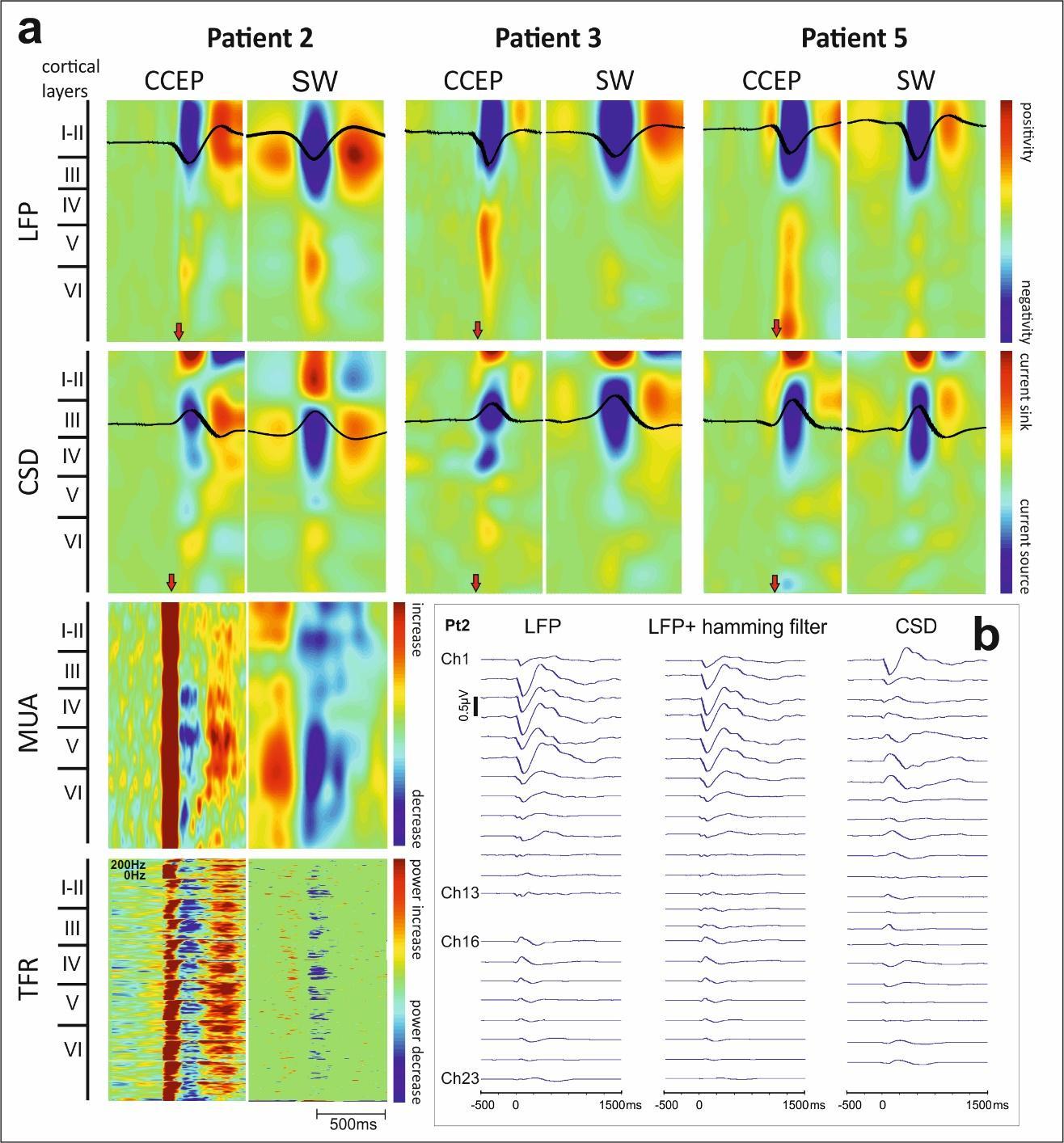


**Supplementary Figure S5.** **Normalized field potential and current source amplitude values of N2 and slow wave downstate (SWd), detected on each microelectrode-contact in four patients, with histological data.**

Multicolor bar indicates different cortical layers. The intra-cortical appearance pattern of N2 and SWd shows only minor differences. In all four patients the maximal amplitudes of field potentials are detected in upper cortical layers. Distribution of current sources shows upper and middle layer predominance.


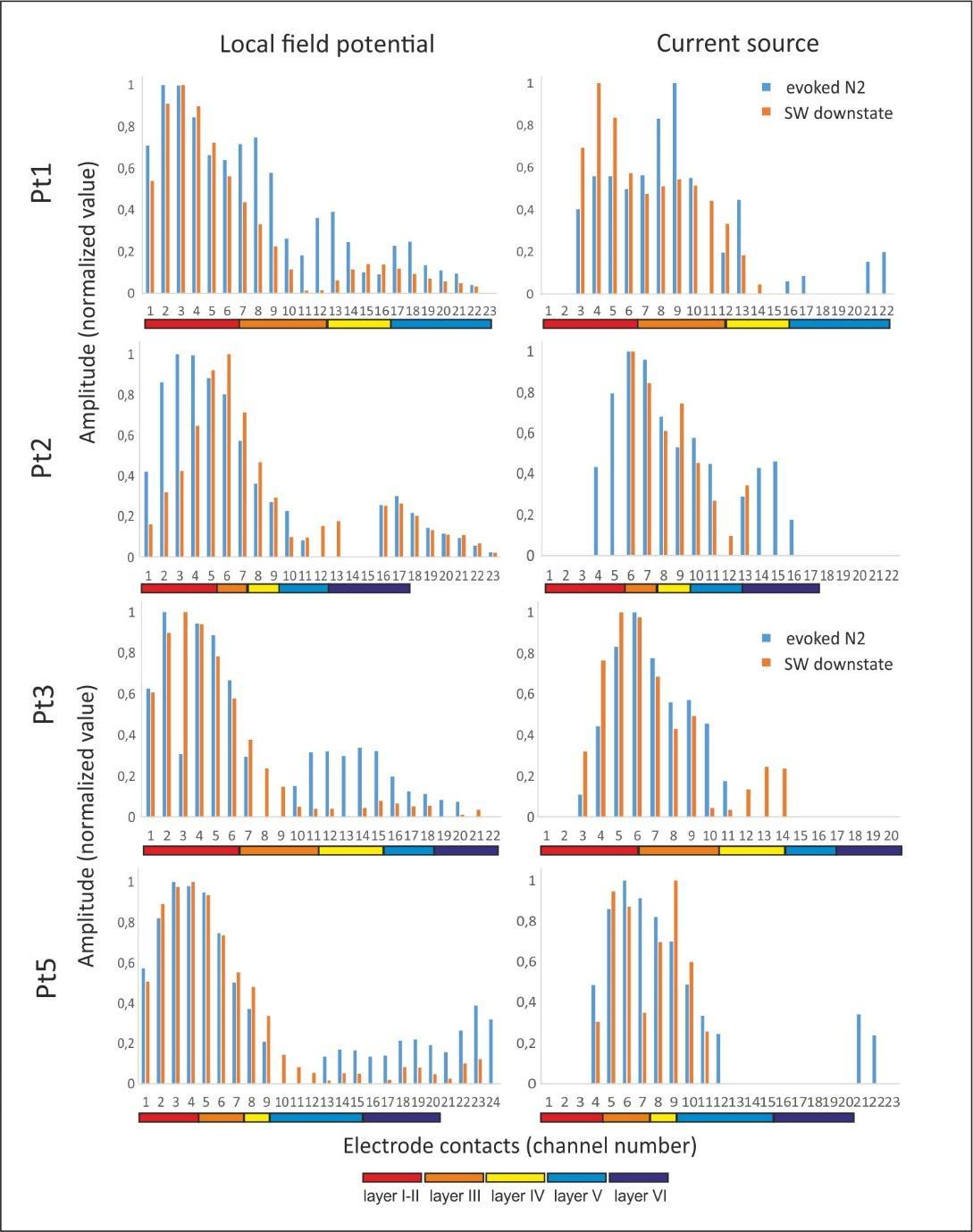


**Supplementary Figure S6. Representative examples of autocorrelations of clustered single-units from each layer.**

Each panel shows the autocorrelation of one unit clustered from the layer shown on the right side of the figure in a window of 100 ms. Insets show the spike morphology of each unit.

**
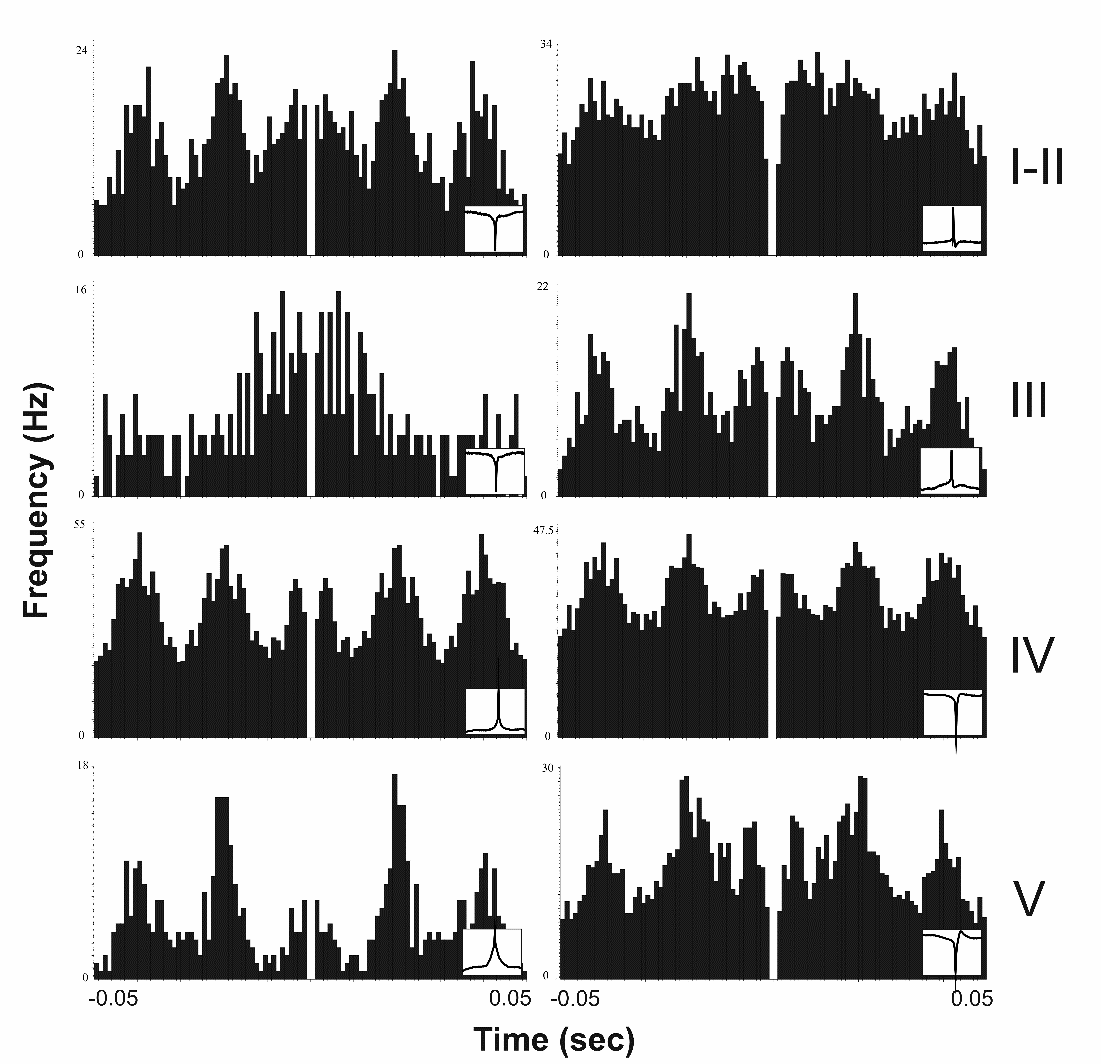
**
